# PREgnancy Care Integrating translational Science, Everywhere (PRECISE): a prospective cohort study of African pregnant and non-pregnant women to investigate placental disorders – cohort profile

**DOI:** 10.1136/bmjopen-2024-091831

**Published:** 2025-05-11

**Authors:** Rachel Craik, Joseph Akuze, Marie-Laure Volvert, Hannah Blencowe, Moses Mukhanya, Prestige Tatenda Makanga, Corssino Tchavana, Sophie E Moore, Anifa Vala, Angela Koech, Rachel M Tribe, Alison Noble, Baboucar Bah, Umberto D’Alessandro, Marianne Vidler, Domena Tu, Sonia Maculuve, Onesmus Wanje, Yahaya Idris, Grace Mwashigadi, Marvin Ochieng, Veronique Filippi, Anna Roca, Laura A Magee, Lucilla Poston, Hiten D Mistry, Yorro Bah, Jing Li, Marleen Temmerman, Esperanca Sevene, Hawanatu Jah, Emily Mwadime, Ben Barratt, Aris T Papageorghiou, Liberty Makacha, Lazaro Quimice, Fatima Touray, Tatiana Salisbury, Fatoumata Kongira, Peter von Dadelszen, the PRECISE Network, Patricia Okiro

**Affiliations:** 1 Department of Women and Children’s Health, School of Life Course and Population Sciences, Faculty of Life Sciences and Medicine, King’s College London, London, UK; 2 Nuffield Department of Women’s & Reproductive Health, University of Oxford, Oxford, UK; 3 Centre for Maternal Reproductive Adolescent and Child Health (MARCH), London School of Hygiene & Tropical Medicine, London, UK; 4 Centre of Excellence in Women & Child Health, The Aga Khan University, Nairobi, Kenya; 5 Department of Surveying and Geomatics, Midlands State University, Gweru, Zimbabwe; 6 Centro de Investigação em Saúde de Manhiça, Manhiça, Mozambique; 7 Department of Engineering, Institute of Biomedical Engineering, University of Oxford, Oxford, UK; 8 MRC Unit The Gambia at the London School of Hygiene and Tropical Medicine, Fajara, The Gambia; 9 Obstetrics & Gynaecology, The University of British Columbia, Vancouver, British Columbia, Canada; 10 Department of Obstetrics and Gynaecology and BC Children’s Hospital Research Institute, The University of British Columbia, Vancouver, British Columbia, Canada; 11 MRC Unit The Gambia at the London School of Hygiene and Tropical Medicine, Banjul, The Gambia; 12 International Centre for Reproductive Health, Ghent, Belgium; 13 Department of Physiological Sciences, Clinical Pharmacology, Faculdade de Medicina, Universidade Eduardo Mondlane, Maputo, Mozambique; 14 Environmental Exposures & Public Health, Imperial College London, London, UK; 15 Department of Health Service and Population Research, Institute of Psychiatry, Psychology and Neuroscience, King’s College London, London, UK

**Keywords:** Pregnant Women, REPRODUCTIVE MEDICINE, PUBLIC HEALTH

## Abstract

**Abstract:**

**Purpose:**

The PREgnancy Care Integrating translational Science, Everywhere Network was established to investigate specific placental disorders (pregnancy hypertension, preterm birth, fetal growth restriction and stillbirth) in sub-Saharan Africa. We created a repository of clinical and social data with associated biological samples from pregnant and non-pregnant women. Alongside this, local infrastructure and expertise in the field of maternal and child health research were enhanced.

**Participants:**

Pregnant women were recruited in participating health facilities in The Gambia, Kenya and Mozambique at their first antenatal visit or at the time a placental disorder was diagnosed (Kenya and The Gambia only). Follow-up study visits were conducted in the third trimester, delivery and 6 weeks to 6 months postpartum. To elucidate the difference between pregnancy and non-pregnancy biology in these settings, non-pregnant nulliparous and parous women, aged 16–49 years, were recruited opportunistically primarily from family planning clinics in Kenya and Mozambique, and randomly through the Health and Demographic Surveillance System in The Gambia. Non-pregnant participants only had one study visit. Biological samples were processed rapidly and locally, stored initially in liquid nitrogen and then at −80°C, and details entered into an OpenSpecimen database linked to their social determinants and clinical research data.

**Findings to date:**

A total of 6932 pregnant and 1825 non-pregnant women were recruited to the study, providing a repository of clinical and social data and a biorepository of 482 448 samples. To date, baseline descriptive analysis of the cohort has been undertaken, as well as a substudy on the prevalence of COVID-19 in the cohort.

**Future plans:**

Analysis of data and samples will include an analysis of biomarker and social and physical determinants of health and how these interact in a systemic approach to understanding the origins of common placental disorders. The data from non-pregnant women will provide control data for comparison with the data from normal and complicated pregnancies. Findings will be disseminated to local stakeholders and communities through meetings and ongoing community engagement and globally by publication and presentations at scientific meetings.

**STRENGTHS AND LIMITATIONS OF THIS STUDY**

- The study is unique as it enables investigation of the drivers and the impact of placental disorders in over 6900 women in three geographically diverse settings in sub-Saharan Africa, before, during and following the COVID-19 pandemic.
- Information regarding women’s social and physical environment, including nutrition, air quality, water, sanitation and hygiene, as well as clinical data (medical history and pregnancy information), was collected in a similar manner in all three sites.
- The PREgnancy Care Integrating translational Science, Everywhere (PRECISE) study has been extended to follow mothers and their children up to 4 years after delivery, focusing on both maternal and child health trajectories (PRECISE-DYAD study).
- Women were recruited from healthcare facilities in a single geographic region in each of the three countries, and therefore, results may not be generalisable beyond those regions.

## Introduction

 Despite international efforts, women and their infants in low-income and middle-income countries (LMICs) remain disproportionately affected by poor pregnancy outcomes.^1^ Over half of the reported global burden of maternal deaths occurs in sub-Saharan Africa, with the leading causes being haemorrhage and hypertensive disease.^2 3^ In 2021, an estimated 1.9 million pregnancies ended in stillbirth, with a further 2.3 million liveborn children dying during the first month of life. The recent Lancet Vulnerable Newborn Series reported that 26.2% of live births globally are born preterm and/or small for gestational age (SGA) and that these small vulnerable babies account for 55% of all neonatal deaths.^4^ Over 75% of these adverse outcomes occur in LMICs, with the highest rates in sub-Saharan Africa and South Asia; regions with the least research into pathways relating to poor pregnancy outcomes.

The PREgnancy Care Integrating translational Science, Everywhere (PRECISE) Network (https://precisenetwork.org) was created to address these disparities in pregnancy outcomes for women and children in LMICs. PRECISE aims to better understand the biological pathways leading to pregnancy complications in sub-Saharan Africa, to inform future interventions and optimise outcomes.

The PRECISE study is a prospective observational study comprising a cohort of pregnant women and non-pregnant women of reproductive age in The Gambia, Kenya and Mozambique. Purposively, the protocol was designed to recruit a cohort of both urban and rural-dwelling women. Extensive data and biological samples were collected from each participant to enable detailed investigation of the pathways to placental disorders, with a focus on pregnancy hypertension, foetal growth restriction (FGR), preterm birth and stillbirth. The study data and biorepository will underpin future investigation of the additional challenges to optimal pregnancy outcomes and well-being, including poor nutrition, infectious and non-communicable diseases, adverse environmental factors and familial and societal expectations leading to a lack of independent decision-making around their own pregnancy.^5^,^8^ The non-pregnant cohort will provide culturally, ethnically and spatially relevant control data with which to compare women with normal and complicated pregnancies. Studies of PRECISE children will evaluate the longer-term sequelae of exposure in utero to maternal placental disorders and also provide a unique opportunity to assess the impact of suboptimal social and physical exposures in pregnancy on the health of children in these settings.

In this paper, we summarise the characteristics of the recruited PRECISE study and highlight the potential role the data-linked biorepository may have in guiding prevention, screening and treatments for pregnant sub-Saharan African women and their children.

## Cohort description

### Study setting

The PRECISE Network study was undertaken in three sub-Saharan African countries in collaboration with the MRC Unit The Gambia at the London School of Hygiene and Tropical Medicine, The Gambia, the Aga Khan University in East Africa, Kenya, and the Centro de Investigação de Saúde de Manhiça (CISM), Mozambique.

In The Gambia, the study was conducted in the district of Farafenni, Central Gambia, on the Transgambian road linking Dakar with Ziguinchor in southern Senegal. The recruiting centres were Farafenni District Hospital (urban) and two rural primary healthcare facilities in Illiasa and Nyagen Sanjal. In Kenya, recruiting centres were Mariakani Sub-County Hospital (urban) in Kaloleni subcounty and Rabai Subcounty Hospital (rural) in Rabai subcounty. Both hospitals are located in Kilifi county in coastal Kenya. In Mozambique, recruiting centres were Manhiça District Hospital (primarily urban population) and in Xinavane Rural Hospital (primarily rural population) in Maputo Province, Southern Mozambique. Women’s residence was captured at each hospital visit, and their villages were classified as urban, periurban or rural, based on the Global Human Settlement Layer.^9^

### Patient and public involvement

The PRECISE Network concept and design was informed by decades-long collaborations with patient advocacy organisations such as the Preeclampsia Foundation (USA and Canada), Action on Pre-eclampsia (UK, Australia and New Zealand), Zuri Nzilani (Kenya) and Sands, the Stillbirth and Neonatal Death Charity (UK).

Specifically, for the PRECISE Network, the PRECISE Network recruiting sites have been working and built trust with the participating communities for many years. The local PRECISE leadership and study teams informed participating communities of the study and, particularly, had in-depth discussions on the best approach within a given community for the collection of samples that may have cultural or religious significance (eg, maternal blood, cord blood and placental samples). In addition, the teams discussed with communities the best approach for collecting specimens immediately following childbirth (cord blood and placental tissue) and returned the placenta to the family, if that was their wish, after it was weighed and photographed and small samples were taken from it. Community engagement activities were conducted repeatedly in all study sites to ensure that the women, and communities in which they live, were aware of the PRECISE programme of work. In Mozambique, 97 meetings were held with 2163 participants, in The Gambia, 175 meetings were held involving over 2300 participants, and in Kenya, 97 meetings were held with 2403 participants.

Once the study results have been published, participants will be informed of the results through the PRECISE website (https://precisenetwork.org/) and will be sent details of the results using infographics suitable for a non-specialist audience.

### Study design

The PRECISE study was a prospective observational cohort study with recruitment between June 2019 and December 2022, with a pause in recruitment in April–July 2020 due to the COVID-19 pandemic.

The study protocol has been described in detail elsewhere.^10^,^12^ Briefly, three groups were enrolled.

#### Unselected pregnancy cohort (UNS)

Pregnant women planning to give birth in a health facility within the study area were recruited when attending for routine antenatal care (ANC). There were four scheduled study visits, two antenatal visits (the first at the ANC booking visit and the second during the third trimester, at least 8 weeks after the first visit), a visit during delivery and a final visit between 6 weeks and 6 months postpartum. Where women did not give birth in the hospital, but attended immediately after delivery, delivery data were collected postpartum. Data at unscheduled visits due to any health concerns were recorded by the study team to identify the onset of any complications. Study visits were aligned with standard ANC/postpartum visits to both minimise the burden on study participants and to facilitate the collection of routinely collected data.

#### Time of disease cohort (ToD)

Recruited in The Gambia and Kenya, it included pregnant women not included in the UNS and attending the study health facilities for hypertension, suspected FGR or a stillbirth. The study visits followed the same schedule as those in the UNS cohort, depending on the stage of pregnancy and the time of recruitment.

#### Women of reproductive age (WRA)

Each site recruited non-pregnant women as a regionally relevant non-pregnancy control group. To capture the seasonal variation in both environmental exposures and infectious diseases, approximately 50 women per month were recruited. In Kenya and Mozambique, both nulliparous (6.4% and 9.9%, respectively) and parous women were recruited opportunistically at family planning clinics, while in The Gambia, nulliparous (28.7%) and parous women were selected randomly from the local Health and Demographic Surveillance System and then visited at home by field staff. Consenting women participated in a single study visit.

As recruitment occurred over a period of 42 months, some women were initially in the WRA cohort but then became pregnant and were recruited to the UNS cohort, and vice versa. In addition, women could be recruited to the pregnancy cohorts again with any subsequent pregnancy.

### Study population

**Figure 1:**
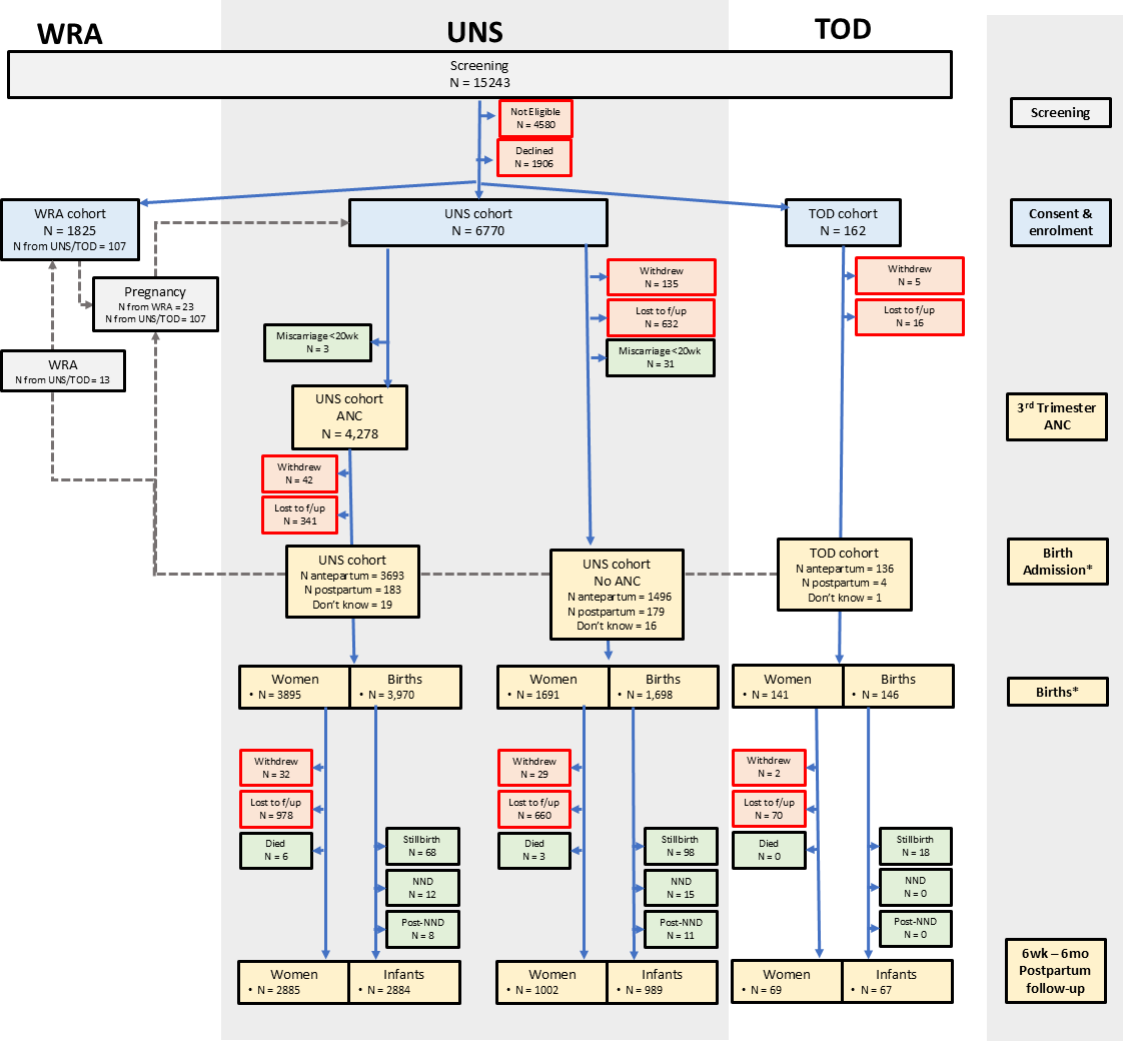
Flow diagram of participants in the PRECISE cohort. Some of the postpartum follow-up visits in Kenya and The Gambia were conducted as part of the PRECISE-DYAD study. All the data collected in the PRECISE postpartum visit were collected at the DYAD visit. ANC, antenatal care; mo, months; NND, neonatal death; PRECISE, PREgnancy Care Integrating translational Science, Everywhere; TOD, time of disease; UNS, unselected pregnancy; wk, weeks; WRA, women of reproductive age. *Eighteen pregnancy outcome could not be classified.

Overall, 6932 pregnant women and 1825 non-pregnant women were recruited. Figure 1 shows the flow of participants through the study. Site-specific information is provided in online supplemental figure S1 for Kenya, online supplemental figure S2 for Mozambique and online supplemental figure S3 for The Gambia. Of the 6932 pregnant women, 4277 had a visit in the third trimester and delivery outcome data were captured for 5735 (82.7%) participants.

Across all sites, 107 women were recruited to the UNS cohort two times, 13 women entered the WRA cohort after being in the UNS cohort and 23 women from the WRA cohort then entered the UNS cohort. 214 (2.4%) women withdrew consent to participate.

### Data collection

Clinical data were collected on Android tablets by research staff at the healthcare facilities through interviews with the participants and from their medical records; biological samples were collected at PRECISE visits. The data collected at each visit are provided in table 1.

**Table 1:** Summary of data and samples collected in PRECISE visits

	Women of reproductive age	Pregnancy cohorts			
		Visit 1 (booking)	Visit 2 (3rd trimester)	Visit 3 (delivery)	Visit 4 (6 weeks to 6 months postpartum)
Clinical data					
General visit information	●	●	●	●	●
Medical history	●	●	–	–	–
Pregnancy information	–	●	–	–	–
Medication	●	●	●	–	–
Clinical assessment	●	●	●	●	●
Investigations and complications	●	●	●	–	–
COVID-19 (since July 2021 except Kenya)	●	●	●	●	●
Maternal outcomes	–	–	–	●	–
Infant outcomes	–	–	–	●	●
Laboratory information	●	●	●	●	●
Non-clinical data					
Maternal demographics	●	●	–	–	–
Environment and WASH	●	●	–	–	–
Nutrition	●	●	–	–	–
Health and disability (WHODAS)	–	●	–	–	●
Clinical assessments					
Vital signs (BP, HR, Pulse Ox, Haem, RR)	●	●	●	●	●
Anthropometry (height, weight, leg length, MUAC)	●	●	●	●	●
Pregnancy dating	–	●	–	–	–
Biological samples					
Maternal blood	●	●	●	●	●
Urine	●	●	●	●	●
Vaginal swab	●	●	–	●	–
Cord blood/placenta	–	–	–	●	–
Infant heel prick	–	–	–	●	●

BP, blood pressure; Haem, haemoglobin; HR, heart rate; MUAC, mid-upper arm circumference; PRECISE, PREgnancy Care Integrating translational Science, Everywhere; Pulse Ox, pulse oximetry; RR, Respiratory Rate; WASH, Water, Sanitation, and HygieneWHODAS - ; WHODAS, World Health Organization Disability Assessment Schedule.

During the COVID-19 pandemic, when research activities were paused, a minimal dataset was collected from women where possible. This included the date of birth, birth weight, sex of the baby and the outcomes for the mother and baby.

### Gestational age (GA) estimation

Accurate pregnancy dating relies on early pregnancy ANC, optimal with ultrasound. In the Gambian and Kenyan sites, similar to many other LMIC settings, coverage of this early pregnancy ultrasound in routine ANC is low. In these settings, dating of pregnancies usually relies on last menstrual period (LMP) and/or symphyseal fundal height (SFH). To overcome this challenge in these two sites, the TraCer GA device was used.^13^ This device uses a commercially available battery-powered wireless ultrasound probe (Konted) and is connected through Wi-Fi to the Android tablet in which the TraCer app is installed. This app is designed for use with minimal training; healthcare workers were trained to capture three videos of the foetal head to enable measurement of the foetal head circumference (HC) and transcerebellar diameter (TCD). The videos were sent to the ultrasound team and reviewed by an experienced sonographer, measuring the HC and TCD to calculate the GA. As this is currently a research tool, TraCer-estimated GA was not used for clinical care. Prior to implementation in PRECISE, a qualitative acceptability and usability study was conducted in Kenya, where the device was found to be well received by both healthcare workers and pregnant women.^14^ In addition to the TraCer data, in The Gambia and Kenya, LMP and SFH were recorded together with ultrasound scan information when available.

In contrast, pregnancy ultrasound scans were routinely available in both participating hospitals in Mozambique, with 83.2% of recruits receiving an ultrasound scan for pregnancy dating during routine ANC. Therefore, TraCer was not used in Mozambican recruiting centres.

For each woman, GA was assessed using a hierarchy based on the reliability of the method used, with routine ultrasound-based dating being prioritised if available. When foetal ultrasound measurements were available, GA was calculated from the most accurate method, namely foetal crown rump length before 14 weeks,^15^ or HC and femur length using the INTERGROWTH standards between 14 and 24 weeks^16^; if not available, GA was calculated from the estimated due date based on available routine ultrasound. When ultrasound was unavailable, the TraCer app was used to estimate GA based on foetal HC and TCD, and if TraCer was unavailable, GA estimates were based on LMP. Finally, estimates based on SFH were only used for women with no other method available, as this appears inferior to the methods above.^17^ GA at delivery of ≥43^+0^ weeks was considered implausible. For any given method, where GA at delivery was ≥43^+0^ weeks, the estimate was excluded and the estimate for the next method from the hierarchy was preferentially used.

Since LMP relies on recall, which may be inaccurate, we reviewed all participants dated by LMP to identify potential errors. Where the GA by LMP and SFH differed by more than 14 days, we introduced birth weight as a guide to accuracy; LMP was considered plausible where the birth weight fell within 2 SD of the 50th centile of birth weight for GA and sex, this was used for final GA assessment. Otherwise, the SFH-based estimate was assessed to determine if this yielded a more plausible GA estimate (see online supplemental figure S4 for full details). A summary of pregnancies dated by each method is outlined in table 2.

**Table 2:** Method of gestational age assignment by country

	Kenya	Mozambique	The Gambia	Total
US (EDD by biometry measurements)*	248 (9.0%)	1301 (69.2%)	9 (0.7%)	1558 (26.6%)
US (EDD by US machine)	68 (2.5%)	346 (18.4%)	367 (30.4%)	781 (13.4%)
TraCer	818 (29.7%)	0 (0.0%)	309 (25.6%)	1127 (19.3%)
LMP	1174 (42.6%)	190 (10.1%)	280 (23.2%)	1644 (28.1%)
SFH	391 (14.2%)	36 (1.9%)	199 (16.5%)	626 (10.7%)
No GA assigned	38 (1.4%)	3 (0.2%)	30 (2.5%)	71 (1.2%)
Outliers	22 (0.8%)	4 (0.2%)	15 (1.2%)	41 (0.7%)

* Biometry measurements were crown rump length or head circumference and femur length.

EDD, Estimated Date of Delivery; GA, gestational age; LMP, last menstrual period; SFH, symphyseal fundal height; US, ultrasound.

### Data and sample management

Data management was undertaken by the co-ordinating team at King’s College London and the University of British Columbia, and the in-country teams. In each country, a data manager was responsible for the local database, including data entry and cleaning. A data manager in the co-ordinating team had oversight of all three site databases, including building and installing the database on local servers, running data queries and extracting data for analysis. Data cleaning and monitoring were undertaken at local and central levels. The local data managers were responsible for running their own queries to identify outliers, and missing or inconsistent data. In addition, the central team sent monthly reports to the local data managers with any additional data queries. These processes optimised data accuracy.

All data were collected through electronic data capture (EDC), via tablets during study visits. Initially, Research Electronic Data Capture (REDCap), a secure, web-based software platform,^18 19^ was used for EDC in all three sites; however, the Gambian and Kenyan teams experienced challenges with synchronising data in the REDCap database, due to the complexity of the database. To overcome this challenge, the team built the database on the ODK-x (Open Data Kit XForms) platform,^20^ which was used for EDC from February 2020. All databases had in-built validation and programming rules to implement skip logics and cross-validation rules. Range limits were set to minimise data entry errors.

Initially, BAOBAB^21^ was used for laboratory information management. However, this system requires a reliable internet connection for data entry, which was not always available. To overcome this, a second open source system was built on the OpenSpecimen platform and used from February 2020.^22^ OpenSpecimen was configured with in-built validation to minimise errors when data were collected, as well as having an ‘offline’ data collection tool to capture data when there was no internet. Offline captured data were uploaded subsequently to the OpenSpecimen platform when internet access was available.

All data and samples are the property of the country teams in which the participants resided during their participation. No identifiers are ever shared outside the country. Each recruiting country team maintains an updated version of their own dataset, and any queries are run through them; updates are shared between the country database and the linked database with data from the three countries (currently at King’s College London). Women are identified by a unique study ID that is shared between the social determinants and clinical database and the laboratory information system.

### Biological samples

PRECISE has created an extensive biorepository, including maternal blood (blood spots, buffy coat, plasma and serum), urine, vaginal swabs, placenta biopsies, cord blood and infant heel pricks. The number of women providing samples at each study visit is shown in table 3. Details of the samples collected can be found in online supplemental table S1. All samples were processed promptly in temperature-regulated laboratories and, when appropriate (eg, plasma, serum, whole blood, urine, placenta), initially placed in liquid nitrogen tanks for initial storage and subsequent transport to −80°C freezers in central facilities in Banjul (The Gambia), Nairobi (Kenya) and Manhiça (Mozambique).^10^

**Table 3:** Number of women with samples collected by study visit

	WRA	Pregnancy cohorts			
	Visit 1	Visit 1	Visit 2	Delivery	Postpartum
Maternal blood	1720	6874	4215	4412	2420
Urine	1741	6850	4331	4141	2892
Vaginal swabs	1098	5329		3136	
Placental/membrane/cord samples				3617	
Cord blood				4023	

WRA, women of reproductive age.

An initial quality control check was performed on 30 vaginal swabs, 29 cord blood buffy coats and 30 placental tissue samples for each of the three sites. Samples were selected by their processing time (ie, time from collection to the freezer), including the five samples with the shortest processing time and five samples with the longest processing time, for each country and sample type. DNA was extracted from each sample, quantified and run on gel electrophoresis to assess evidence of degradation. Overall, sample qualities were high, with 87% of the samples (97% vaginal swabs, 71% cord blood buffy coat and 73% placental samples) having the recommended 260/280 ratio for downstream genetic analysis. The mean sample processing time was 2.78 hours (95% CI 2.30 to 3.25), which has no correlation with the sample DNA concentration or 260/280 ratio (manuscript in preparation).

## Findings to date

### Characteristics of study participants

The study participants’ baseline characteristics are shown in table 4 and participants’ baseline characteristics by cohort are shown in online supplemental table S2. For both the pregnant and non-pregnant cohorts, demographic differences were observed between the three countries. Marital status varied between sites, with the number of women reported as cohabiting or married being substantially higher in Kenya and The Gambia (91.6% and 97.8%, respectively, for pregnant participants and 82.6% and 81.3% for non-pregnant participants) than in Mozambique (56.2% and 48.8%). In Kenya and Mozambique, approximately 90% of women had received at least a primary school education, while in The Gambia, 62.9% of the women had no formal schooling, possibly due to attendance at Koranic (Arabic) School that is not recognised as formal schooling (consistent with practice for international comparisons). Over half of all pregnant women in all sites were housewives, ranging from 54.8% in Kenya to 87.8% in The Gambia. In terms of religion, the Gambian women were almost exclusively Muslim, the Mozambican participants predominantly Christian and the Kenyan cohort more mixed (around 40% Muslim, 60% Christian). Similarly, parity varied, with almost a quarter of participants from The Gambia being grand multiparous (five or more previous births) compared with fewer than 10% in the other sites.

**Table 4:** Characteristics of study participants by country

	Kenya		Mozambique		The Gambia		All sites	
	WRA	Pregnancy cohort	WRA	Pregnancy cohort	WRA	Pregnancy cohort	WRA	Pregnancy cohort
Number	n=609	n=3584	n=606	n=2097	n=610	n=1251	n=1825	n=6932
Age, median (IQR)	27.0 (23.0, 33.0)	26.0 (23.0, 31.0)	28.0 (23.0, 35.0)	23.0 (19.0, 29.0)	28.0 (22.0, 36.0)	26.0 (22.0, 31.0)	28.0 (23.0, 34.0)	25.0 (21.0, 30.0)
Age at enrolment								
15–19	25 (4.1%)	252 (7.0%)	48 (7.9%)	554 (26.4%)	67 (11.0%)	156 (12.5%)	140 (7.7%)	962 (13.9%)
20–24	192 (31.5%)	1114 (31.1%)	141 (23.3%)	626 (29.9%)	145 (23.8%)	333 (26.6%)	478 (26.2%)	2073 (29.9%)
25–29	158 (25.9%)	1038 (29.0%)	161 (26.6%)	455 (21.7%)	107 (17.5%)	362 (28.9%)	426 (23.3%)	1855 (26.8%)
30–34	117 (19.2%)	733 (20.5%)	104 (17.2%)	270 (12.9%)	104 (17.0%)	223 (17.8%)	325 (17.8%)	1226 (17.7%)
35–39	70 (11.5%)	358 (10.0%)	93 (15.3%)	145 (6.9%)	77 (12.6%)	124 (9.9%)	240 (13.2%)	627 (9.0%)
40–44	34 (5.6%)	81 (2.3%)	43 (7.1%)	42 (2.0%)	64 (10.5%)	45 (3.6%)	141 (7.7%)	168 (2.4%)
45–49	13 (2.1%)	5 (0.1%)	16 (2.6%)	5 (0.2%)	35 (5.7%)	4 (0.3%)	64 (3.5%)	14 (0.2%)
Missing	0 (0.0%)	3 (0.1%)	0 (0.0%)	0 (0.0%)	11 (1.8%)	4 (0.3%)	11 (0.6%)	7 (0.1%)
Gestational age at enrolment		3530 (98.5%)		2097 (100.0%)		1245 (99.5%)		6872 (99.3%)
Median (IQR)		21.7 (16.6, 26.4)		19.4 (15.4, 23.4		21.4 (15.4, 27)		20.9 (16.0, 25.6)
Missing GA		54 (1.5%)		0 (0.0%)		6 (0.5%)		60 (0.8%)
Marital status								
Never married (or single)	55 (9.0%)	220 (6.1%)	293 (48.3%)	906 (43.2%)	97 (15.9%)	22 (1.8%)	445 (24.4%)	1148 (16.6%)
Married/cohabiting†	503 (82.6%)	3282 (91.6%)	296 (48.8%)	1179 (56.2%)	496 (81.3%)	1223 (97.8%)	1295 (71.0%)	5684 (82.0%)
Separated/divorced	42 (6.9%)	55 (1.5%)	4 (0.7%)	7 (0.3%)	12 (2.0%)	6 (0.5%)	58 (3.2%)	68 (1.0%)
Widowed/missing	9 (1.5%)	27 (0.8%)	13 (2.1%)	5 (0.2%)	5 (0.8%)	0 (0.0%)	27 (1.5%)	32 (0.5%)
Highest education level								
None	62 (10.2%)	337 (9.4%)	65 (10.7%)	109 (5.2%)	373 (61.1%)	797 (63.7%)	500 (27.4%)	1243 (17.9%)
Primary	367 (60.3%)	1885 (52.6%)	266 (43.9%)	704 (33.6%)	71 (11.6%)	198 (15.8%)	704 (38.6%)	2787 (40.2%)
Secondary	116 (19.0%)	925 (25.8%)	271 (44.7%)	1258 (60.0%)	119 (19.5%)	198 (15.8%)	506 (27.7%)	2381 (34.3%)
Higher	61 (10.0%)	417 (11.6%)	4 (0.7%)	26 (1.2%)	47 (7.7%)	57 (4.6%)	112 (6.1%)	500 (7.2%)
Missing	3 (0.5%)	20 (0.6%)	0 (0.0%)	0 (0.0%)	0 (0.0%)	1 (0.1%)	3 (0.2%)	21 (0.3%)
Occupation								
Housewife	327 (53.7%)	1963 (54.8%)	361 (59.6%)	1524 (72.7%)	453 (74.3%)	1099 (87.8%)	1141 (62.5%)	4586 (66.2%)
Student	21 (3.4%)	74 (2.1%)	68 (11.2%)	344 (16.4%)	62 (10.2%)	13 (1.0%)	151 (8.3%)	431 (6.2%)
Professional	34 (5.6%)	256 (7.1%)	19 (3.1%)	61 (2.9%)	16 (2.6%)	20 (1.6%)	69 (3.8%)	337 (4.9%)
Factory	10 (1.6%)	86 (2.4%)	10 (1.7%)	15 (0.7%)	0 (0.0%)	0 (0.0%)	20 (1.1%)	101 (1.5%)
Large-scale agriculture	4 (0.7%)	4 (0.1%)	8 (1.3%)	20 (1.0%)	6 (1.0%)	9 (0.7%)	18 (1.0%)	33 (0.5%)
Market trader	61 (10.0%)	345 (9.6%)	84 (13.9%)	91 (4.3%)	25 (4.1%)	48 (3.8%)	170 (9.3%)	484 (7.0%)
Construction	2 (0.3%)	2 (0.1%)	0 (0.0%)	1 (<1%)	0 (0.0%)	0 (0.0%)	2 (0.1%)	3 (<1%)
Business	60 (9.9%)	342 (9.5%)	54 (8.9%)	38 (1.8%)	0 (0.0%)	0 (0.0%)	60 (3.3%)	342 (4.9%)
Informal—employment	74 (12.2%)	457 (12.8%)	0 (0.0%)	0 (0.0%)	0 (0.0%)	0 (0.0%)	74 (4.1%)	457 (6.6%)
Other (specify)	13 (2.1%)	33 (0.9%)	0 (0.0%)	0 (0.0%)	48 (7.9%)	61 (4.9%)	115 (6.3%)	132 (1.9%)
Missing	3 (0.5%)	22 (0.6%)	2 (0.3%)	3 (0.1%)	0 (0.0%)	1 (0.1%)	5 (0.3%)	26 (0.4%)
Religion								
Muslim	222 (36.5%)	1386 (38.7%)	11 (1.8%)	27 (1.3%)	602 (98.7%)	1243 (99.4%)	835 (45.8%)	2656 (38.3%)
Christian	379 (62.2%)	2166 (60.4%)	550 (90.8%)	2042 (97.4%)	8 (1.3%)	8 (0.6%)	937 (51.3%)	4216 (60.8%)
Other (specify)	5 (0.8%)	12 (0.3%)	44 (7.3%)	26 (1.2%)	0 (0.0%)	0 (0.0%)	49 (2.7%)	38 (0.5%)
Missing	3 (0.5%)	20 (0.6%)	1 (0.2%)	2 (0.1%)	0 (0.0%)	0 (0.0%)	4 (0.2%)	22 (0.3%)
% likelihood below the USAID extreme poverty line, median (IQR)	2.5 (1.2, 15.4)	2.3 (0.3, 7.4)	9.7 (3.2, 12.9)	5.7 (0.0, 12.9)	25.2 (16.9, 26.2)	25.2 (12.9, 28.9)	12.9 (2.5, 25.2)	5.7 (0.3, 15.4)
% likelihood below the poverty line, median (IQR)	17.8 (6.1, 36.9)	13.9 (4.6, 30)	28.8 (8.5, 31.7)	21.4 (7.2, 31.7)	46.9 (34.8, 56.0)	46.9 (27.9, 58.6)	31.7 (13.9, 51.3)	20.3 (6.1, 36.9)
Maternal BMI, median (IQR)	22.5 (20.1, 25.5)	24.1 (21.5, 27.8)	23.3 (21.1, 26.8)	24.2 (22.1, 27.0)	20.8 (18.5, 24.1)	22.0 (19.8, 24.8)	22.2 (20.0, 25.7)	23.8 (21.3, 27.1)
Maternal BMI								
<18.5	53 (8.7%)	143 (4.0%)	22 (3.6%)	39 (1.9%)	153 (25.1%)	166 (13.3%)	228 (12.5%)	348 (5.0%)
18.5–24.9	376 (61.7%)	1893 (52.8%)	362 (59.7%)	1172 (55.9%)	329 (53.9%)	793 (63.4%)	1067 (58.5%)	3858 (55.7%)
25–29.9	122 (20.0%)	942 (26.3%)	153 (25.2%)	661 (31.5%)	85 (13.9%)	209 (16.7%)	360 (19.7%)	1812 (26.1%)
+30	55 (9.0%)	558 (15.6%)	68 (11.2%)	225 (10.7%)	43 (7.0%)	80 (6.4%)	166 (9.1%)	863 (12.4%)
Missing	3 (0.5%)	48 (1.3%)	1 (0.2%)	0 (0.0%)	0 (0.0%)	3 (0.2%)	4 (0.2%)	51 (0.7%)
Parity								
Zero	39 (6.4%)	1046 (29.2%)	60 (9.9%)	828 (39.5%)	175 (28.7%)	237 (18.9%)	274 (15.0%)	2112 (30.5%)
One	175 (28.7%)	942 (26.3%)	105 (17.3%)	507 (24.2%)	69 (11.3%)	210 (16.8%)	349 (19.1%)	1659 (23.9%)
Two	135 (22.2%)	659 (18.4%)	129 (21.3%)	336 (16.0%)	56 (9.2%)	185 (14.8%)	320 (17.5%)	1179 (17.0%)
Three	94 (15.4%)	413 (11.5%)	123 (20.3%)	244 (11.6%)	73 (12.0%)	155 (12.4%)	290 (15.9%)	812 (11.7%)
Four	58 (9.5%)	247 (6.9%)	103 (17.0%)	123 (5.9%)	58 (9.5%)	168 (13.4%)	219 (12.0%)	538 (7.8%)
Five or more	108 (17.7%)	277 (7.7%)	86 (14.2%)	59 (2.8%)	179 (29.3%)	296 (23.7%)	373 (20.4%)	632 (9.1%)
HIV status								
No	592 (97.2%)	3480 (97.1%)	429 (70.8%)	1855 (88.5%)	610 (100.0%)	1230 (98.3%)	1631 (89.4%)	6565 (94.7%)
Yes	17 (2.8%)	104 (2.9%)	177 (29.2%)	242 (11.5%)	0 (0.0%)	21 (1.7%)	194 (10.6%)	367 (5.3%)
Village—rural and urban index*								
Periurban	296 (48.6%)	1414 (39.5%)	17 (2.8%)	45 (2.1%)	12 (2.0%)	10 (0.8%)	325 (17.8%)	1469 (21.2%)
Rural	5 (0.8%)	220 (6.1%)	210 (34.7%)	858 (40.9%)	409 (67.0%)	750 (60.0%)	624 (34.2%)	1828 (26.4%)
Urban	308 (50.6%)	1949 (54.4%)	379 (62.5%)	1194 (56.9%)	189 (31.0%)	491 (39.2%)	876 (48.0%)	3634 (52.4%)
Missing	0 (0.0%)	1 (<1%)	0 (0.0%)	0 (0.0%)	0 (0.0%)	0 (0.0%)	0 (0.0%)	1 (0.0%)

* 748 in Kenya, 14 in Mozambique and 95 in Gambia records were missing village name/location at visit 1 and the health facility location was used to impute for their rural and urban index.

† Married and cohabiting includes women who are living with a partner and those who are married, including women where they are one of two or more wives.

BMI, body mass index; GA, gestational age; USAID, United State Agency for International Development; WRA, women of reproductive age.

HIV prevalence varied by country, with low rates in The Gambia (non-pregnant cohort 0%, pregnancy cohort 1.6%) and Kenya (2.8% and 2.9%, respectively), compared with high rates in Mozambique (29.2% and 11.6%, respectively). HIV rates are known to be high in Mozambique, and the lower-than-expected prevalence in the pregnant cohort may be explained by ongoing HIV trials in pregnancy at the same health facilities used by the PRECISE study; HIV-positive pregnant women may have enrolled in these trials instead of in the PRECISE study.

Median body mass index (BMI) measured at recruitment was higher for pregnant than non-pregnant women (23.8 vs 22.2 kg/m^2^) as pregnant women were recruited in the second trimester when some pregnancy weight gain had occurred. To mitigate against this, we collected information regarding mid-upper arm circumference and intend to present these data alongside the BMI in a separate analysis to better understand body composition phenotype.

### Pregnancy outcomes

Analysis of key pregnancy outcomes has been undertaken. Maternal pregnancy outcomes are available for 5745 (82.6%) of the participants (table 5a). In the pregnancy cohorts, birth outcome data were collected from 5814 babies, of which 5601 (96.5%) were live births (table 5b). The remaining 184 (3.2%) of these pregnancies ended in a stillbirth, defined as babies not born alive either at ≥20^+0^ weeks’ gestation or ≥500 g.^23^ These rates varied between sites, with the Kenya rate being the lowest with 62 (2.3%), ranging up to 61 (5.1%) in The Gambia. Pregnancy outcomes by cohort are shown in online supplemental table S3a,b.

**Table 5:** (a) Maternal pregnancy outcomes by country. (b) Infant outcomes by country

(a)				
	Kenya*	Mozambique	The Gambia*	Total
Number of women with pregnancy outcome data	2706	1861	1178	5745
Miscarriages (<20 weeks)	5 (0.2%)	17 (1.0%)	12 (1.0%)	34 (0.6%)
Maternal hypertension	574 (21.2%)	288 (15.5%)	349 (29.6%)	1211 (21.1%)
Maternal gestational hypertension	429 (15.9%)	267 (14.4%)	239 (20.3%)	935 (16.3%)
Maternal chronic hypertension	142 (5.3%)	45 (2.4%)	141 (12.0%)	328 (5.7%)
Maternal pre-eclampsia	194 (7.2%)	51 (2.7%)	155 (13.2%)	400 (6.7%)
Missing maternal hypertension information	914 (33.8%)	81 (4.4%)	315 (26.7%)	1310 (22.8%)
Maternal admissions to ICU	0	4	1	5
Maternal deaths	6	0	3	9
Missing pregnancy outcome	7 (0.3%)	1 (0.1%)	3 (0.3%)	11 (0.2%)

(b)				
	Kenya	Mozambique	The Gambia	Total
Number of children with birth outcome data (total birth)	2754	1863	1197	5814
Number of singletons	2648 (96.2%)	1827 (98.1%)	1135 (94.8%)	5610 (96.5%)
Number of twins	106 (3.8%)	36 (1.9%)	62 (5.2%)	204 (3.5%)
Vital status at birth				
Number of live births	2682 (97.4%)	1800 (96.6%)	1126 (94.1%)	5608 (96.5%)
Number of stillbirths (≥20 weeks)	62 (2.3%)	61 (3.3%)	61 (5.1%)	184 (3.2%)
Missing/birth outcome not classified	10 (0.3%)	2 (0.1%)	10 (0.8%)	22 (0.3%)
Mode of delivery				
Unassisted vaginal/cephalic	2156 (78.3%)	1387 (74.4%)	956 (79.9%)	4499 (77.4%)
Operative vaginal	1 (<1%)	138 (7.4%)	13 (1.1%)	152 (2.6%)
Vaginal breech	12 (0.4%)	129 (6.9%)	5 (0.4%)	146 (2.5%)
Caesarean section	419 (15.2%)	194 (10.4%)	34 (2.8%)	647 (11.1%)
Missing mode of delivery	166 (6.0%)	15 (0.8%)	189 (15.8%)	370 (6.4%)
Birth weight				
Median (IQR)	3002.5 (2700.0, 3289.5)	3092.5 (2800.0, 3390.0)	3000.0 (2713.5, 3305.0)	3017.2 (2732.5, 3302.5)
Birth weight categories				
<2500 g	348 (12.6%)	198 (10.6%)	121 (10.1%)	667 (11.5%)
2500–4000 g	2012 (73.1%)	1615 (86.7%)	848 (70.8%)	4475 (77.0%)
>4000 g	46 (1.7%)	31 (1.7%)	9 (0.8%)	86 (1.5%)
Missing birth weight	348 (12.6%)	19 (1.0%)	219 (18.3%)	586 (10.1%)
Gestational age at delivery	2694 (97.8%)	1855 (99.6%)	1152 (96.2%)	5701 (98.1%)
Median (IQR)	39.0 (37.1, 40.4)	39.6 (38.0, 40.9)	39.0 (37.1, 40.4)	39.1 (37.6, 40.6)
Missing GA	60 (2.2%)	8 (0.4%)	45 (3.8%)	113 (1.9%)
Preterm birth categories				
Extremely preterm: <28^+0^ weeks	36 (1.3%)	23 (1.2%)	22 (1.8%)	81 (1.4%)
Very preterm: 28^+0^–31^+6^	60 (2.2%)	31 (1.7%)	35 (2.9%)	126 (2.2%)
Moderate preterm: 32^+0^–33^+6^	97 (3.5%)	36 (1.9%)	31 (2.6%)	164 (2.8%)
Late preterm: 34^+0^–36^+6^	355 (12.9%)	171 (9.2%)	147 (12.3%)	673 (11.6%)
Total preterm (<37^+0^ weeks)	548 (19.9%)	261 (14.0%)	235 (19.6%)	1044 (18.0%)
Size for gestational age†				
Severely small for gestational age (<3rd centile)	177 (6.4%)	142 (7.6%)	81 (6.8%)	400 (6.9%)
Small for gestational age (3rd–10th centile)	273 (9.9%)	203 (10.9%)	115 (9.6%)	591 (10.2%)
Appropriate for gestational age (10th–90th centile)	1610 (58.5%)	1313 (70.5%)	621 (51.9%)	3544 (61.0%)
Large for gestational age (>90th centile)	177 (6.4%)	96 (5.2%)	69 (5.8%)	342 (5.9%)
Extremely large for gestational age (>97th centile)	80 (2.9%)	55 (3.0%)	38 (3.2%)	173 (3.0%)
Missing size for gestational age	437 (15.9%)	54 (2.9%)	273 (22.8%)	764 (13.1%)
Neonatal admission to neonatal unit				
Yes	57 (2.1%)	58 (3.1%)	15 (1.3%)	130 (2.2%)
Missing admission to neonatal unit	463 (16.8%)	82 (4.4%)	299 (25.0%)	844 (14.5%)

* Six (five in Kenya and one in The Gambia) pregnancy outcome could not be classified into either live birth or miscarriage or stillbirth.

† Calculated using the INTERGROWTH-21st charts.

ICU, intensive care unit.

Overall, 21.1% of the participants had hypertension at delivery. GA assessment at delivery is available for 5686 (98.1%) of the babies. The median GA at delivery was 39 weeks and 1 day, with 18% of all births classified as preterm (<37^+0^ weeks). Overall, 921 (17.1%) babies were SGA, defined as <10th centile using the INTERGROWTH standards; 401 (6.9%) of liveborn babies were less than the 3rd centile.^24^ 130 (2.2%) newborns required admission to the neonatal unit. Five women across the three sites were admitted to intensive care units, and there were nine maternal deaths.

### Biological samples

To date, the biorepository has been used for the quality control assessments (above), for SARS-CoV-2 seroprevalence studies in all sites^25 26^ (Mozambique manuscript submitted) (below), for placental growth factor assays (manuscripts in preparation), the MRC (UK)-funded spontaneous preterm birth substudy (manuscripts in preparation), comparing pulse oximetry-based haemoglobin estimates with measured haemoglobin,^27^ urinary proteinuria (manuscripts in preparation), placental and umbilical cord histology (manuscripts in preparation), differential placental gene expression (manuscript in preparation) and laboratory reference intervals for African pregnancies (manuscripts in preparation).

### SARS-CoV-2 pandemic

PRECISE contributed to the PeriCOVID Africa consortium to describe the seroepidemiology of SARS-CoV-2 infection in pregnancy and to define the impact of a SARS-CoV-2 infection during pregnancy.^28^ Each of the Gambian, Kenyan and Mozambican teams conducted a study using PRECISE samples to assess seroprevalence of SARS-CoV-2. A representative selection of serum samples from the biorepository was tested using a qualitative SARS-CoV-2 ELISA kit (Wantai, total antibodies). Positive samples were then retested for anti-SARS-CoV-2 antinucleocapsid antibodies (Euroimmun, ELISA kits, NCP, qualitative, IgG) and antispike protein antibodies (Euroimmun, ELISA kits, QuantiVac; quantitative, IgG). Seroprevalence increased over the study period in all three countries and coincided with the COVID-19 waves. The highest seroprevalence was observed in late 2021 to early 2022, (Kenya: 89.7%, Mozambique: 79.9% and The Gambia: 85.1%), when the Omicron variant was predominant^25 26^ (submitted to *BMC Pregnancy and Childbirth*). These data differ from the official country reports which suggested a lower prevalence, highlighting the benefit of seroprevalence studies for infectious diseases such as SARS-CoV-2.

Analysis of the dataset is underway for the primary outcomes of hypertension, preterm birth and stillbirths, as well as for the small and vulnerable newborns as a combined group. This extensive dataset is being used widely in the network to address numerous research questions. The PRECISE cohort has received funding from the Wellcome Trust to follow-up the cohort (mothers and children) in The Gambia and Kenya up to 3 years post delivery. This will complement and enable better understanding of the impacts of these pregnancy complications on the mother and child’s future physical, mental and, for children, neurodevelopmental health trajectories.

### Publications to date

To describe the Network’s vision and plans, we published a supplement in *Reproductive Health*.^10^,^1229^ In addition, we have undertaken scoping and systematic reviews to learn about pregnancy cohorts with biorepositories in Africa,^32^ the impact of the climate crisis,^33^ cervicovaginal microbiota^34^ and nutrition^35^ (and calcium specifically^36^) on pregnancy outcomes, choice of antihypertensives in pregnancy,^37^ the content of respectful maternity care training packages^38^ and to develop conceptual frameworks for pre-eclampsia and stillbirth^39^,^42^ (including a UNICEF report^43^). Our Zimbabwe-based health geography team has published regarding mobile tools^44^ and mobilising transport to optimise pregnancy outcomes.^45^ We have developed and are testing the TraCer device for low-cost, ultrasound-based and AI-based pregnancy dating^13 14^ and shown that the Masimo Rad-67 pulse oximeter does not accurately measure haemoglobin.^27^ About half of our cohort was seropositive for SARS-CoV-2,^25 26^ which will be important as we follow up the cohort in PRECISE-DYAD.^46 47^ We have assessed postpartum recovery after severe maternal illness.^48^

### Collaboration

We have successfully established the PRECISE database of clinical and social data and a biorepository. Together, these strengthen research capacity in sub-Saharan Africa, while simultaneously investigating the impact of placental conditions in these communities. Our priority is that this resource enables researchers in Africa to use the data and samples to answer relevant contextual research questions and be a vehicle to build careers and local research capacity. To enable this, each site team owns their data and samples.

External and PRECISE network researchers are welcome to request use of data and samples,^10^ the scientific potential of which is then reviewed by the data and samples access committee. We actively promote our preferred model of working, which is to establish collaborations that ensure representative input from country site researchers and local teams (ie, teams who led on recruitment and data/sample collection) in analysis and reporting. This strengthens the relevance of the outputs, as site teams are best placed to interpret the complexities of their context and data. Data requests are directed to the network via an email contact (precise@kcl.ac.uk) and follow established data request procedures thereafter. Our data and sample access processes are described in more detail on the study website.^49^

## Supplementary material

10.1136/bmjopen-2024-091831 online supplemental figure 1

10.1136/bmjopen-2024-091831 online supplemental figure 2

10.1136/bmjopen-2024-091831 online supplemental figure 3 * ^1^

10.1136/bmjopen-2024-091831 online supplemental figure 4

10.1136/bmjopen-2024-091831 online supplemental table 1

## Data Availability

Data are available on reasonable request.
