# Supplementary figures and images for: PREgnancy Care Integrating translational Science, Everywhere (PRECISE): a prospective cohort study of African pregnant and non-pregnant women to investigate placental disorders – cohort profile
Source: BMJ Open. 2025 May 11;15(5):e091831. doi: 10.1136/bmjopen-2024-091831 (PMC12067852; doi:10.1136/bmjopen-2024-091831)

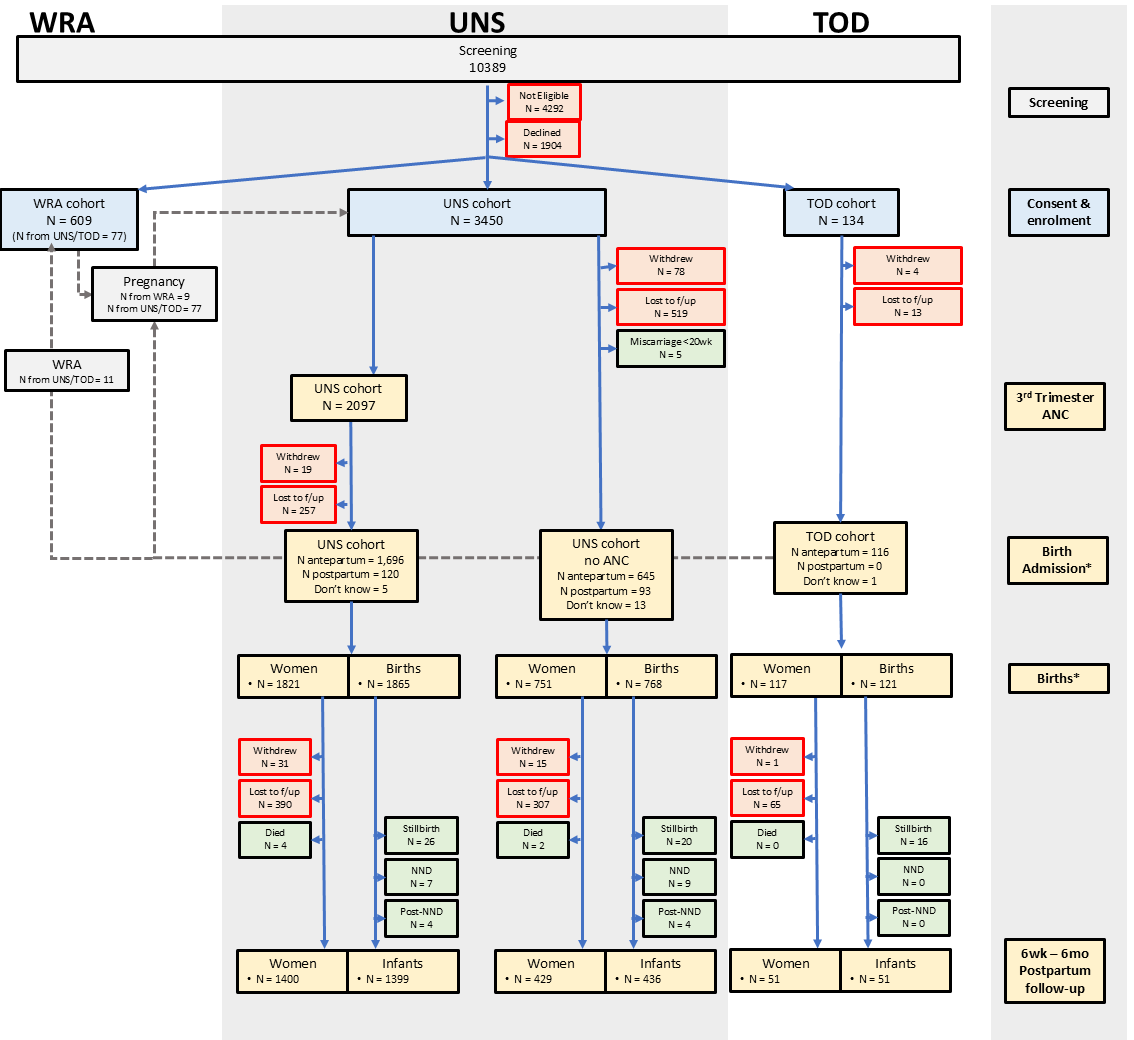

Supplement: online supplemental figure 1 [file bmjopen-15-5-s001.png]

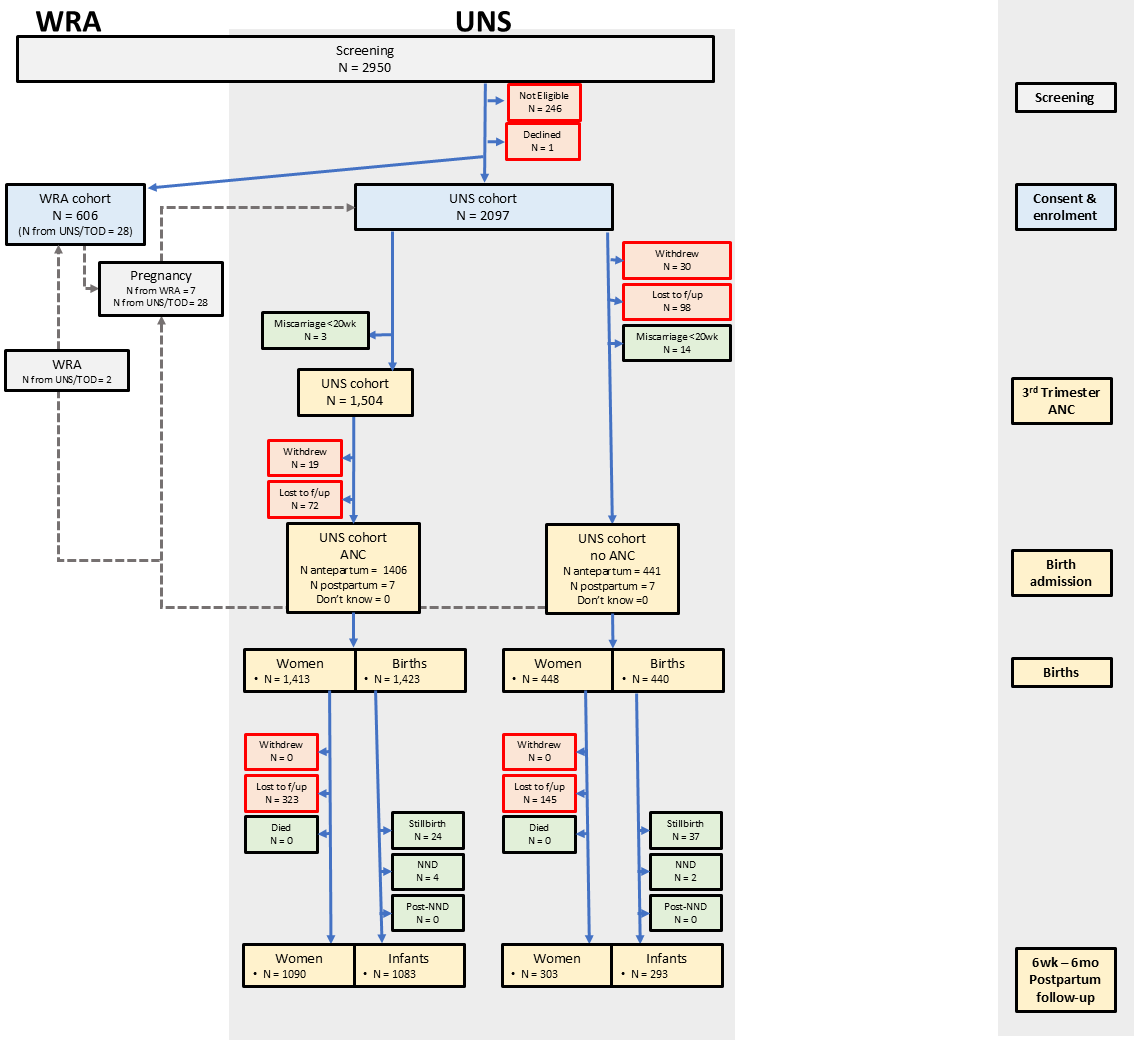

Supplement: online supplemental figure 2 [file bmjopen-15-5-s002.png]

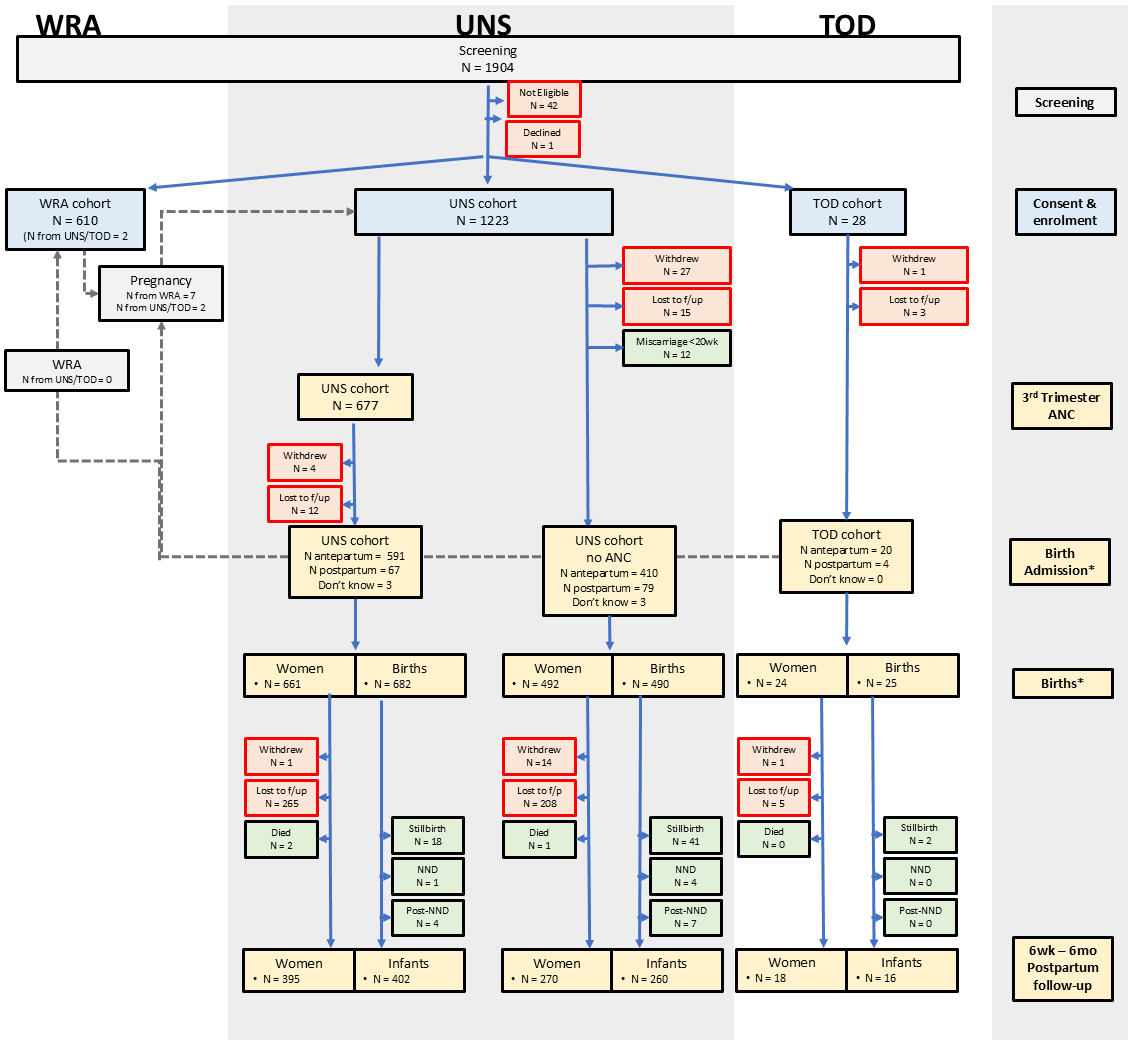

Supplement: online supplemental figure 3 [file bmjopen-15-5-s003.png]

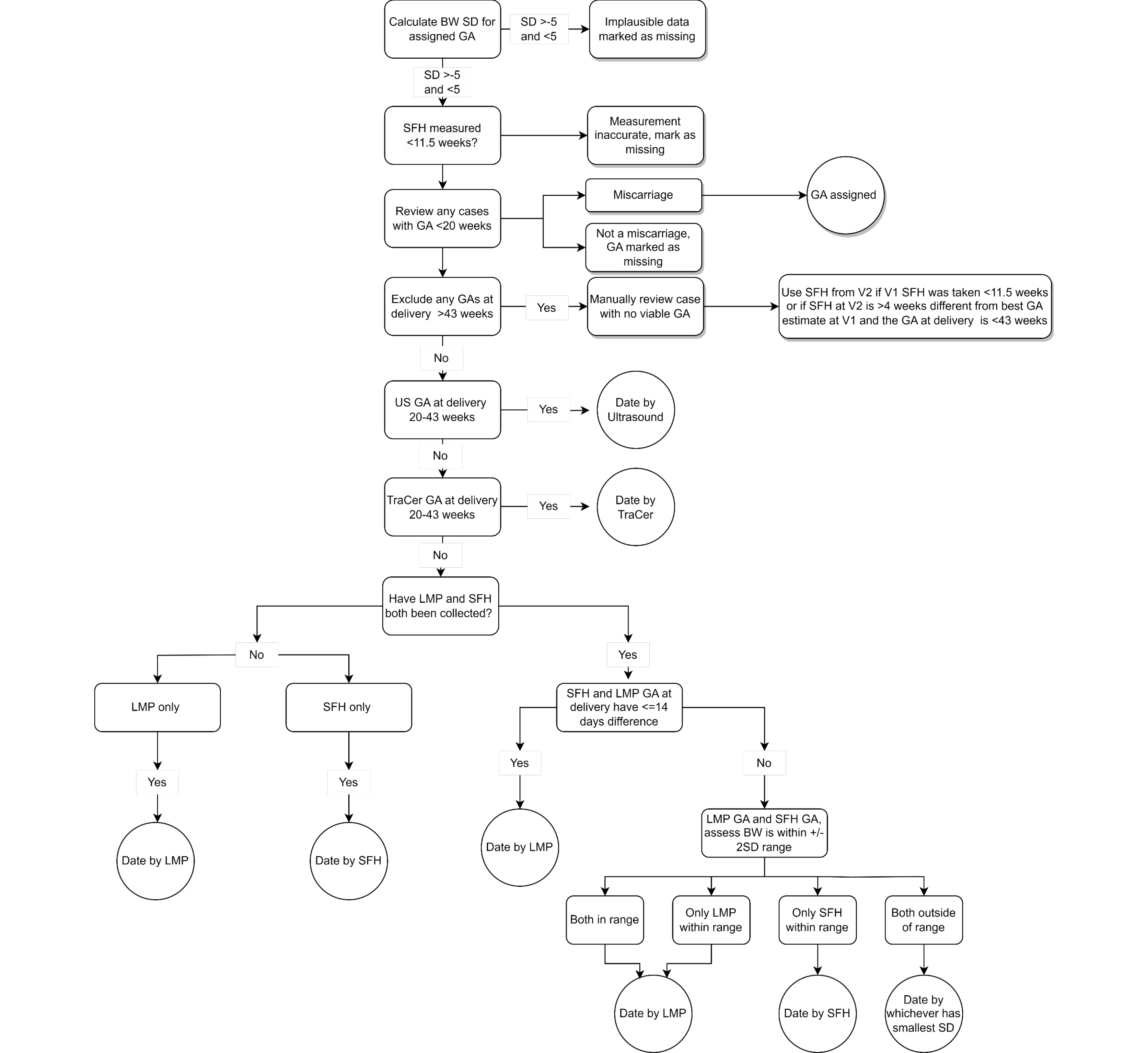

Supplement: online supplemental figure 4 [file bmjopen-15-5-s004.tiff]
