# Supplementary material for: PREgnancy Care Integrating translational Science, Everywhere (PRECISE): a prospective cohort study of African pregnant and non-pregnant women to investigate placental disorders – cohort profile
Source: BMJ Open. 2025 May 11;15(5):e091831. doi: 10.1136/bmjopen-2024-091831 (PMC12067852; doi:10.1136/bmjopen-2024-091831)
Supplement: online supplemental table 1 [file bmjopen-15-5-s005.docx]

**Supplementary information**

**Cohort Profile: PREgnancy Care Integrating translational Scince, Everywhere (PRECISE), a prospective cohort study of pregnant women in The Gambia, Kenya and Mozambique investigating placental disorders**

Table S1: Overview of biological sample collection per participant

TableS2: Characteristics of pregnant participants by cohort by country

Table S3a: Maternal Pregnancy outcomes by cohort and by country

Table S3b: Infant outcomes by cohort and by country

#

Table S1: Overview of biological sample collection per participant

| Pregnant women (cohort 2-5) | Gambia (n=1251) | | | | Kenya (n=3439) | | | | Mozambique (n=2096) | | | |
| --- | --- | --- | --- | --- | --- | --- | --- | --- | --- | --- | --- | --- |
|  | Booking | ANC | Delivery | Postpartum | Booking | ANC | Delivery | Postpartum | Booking | ANC | Delivery | Postpartum |
| Whole blood | 1239 | 676 | 802 | 291 | 3539 | 1882 | 1512 | 1374 | 2095 | 1655 | 1802 | 1364 |
| Blood spot | 1236 |  |  |  | 3419 |  |  |  | 2096 |  |  |  |
| Maternal Serum | 1227 | 671 | 794 | 291 | 3533 | 1883 | 1515 | 1374 | 2095 | 1656 | 2095 | 1362 |
| Maternal Plasma | 1235 | 672 | 801 | 290 | 3534 | 1881 | 1511 | 1372 | 2095 | 1656 | 1805 | 1364 |
| Maternal Buffy coat | 1237 | 676 | 802 | 291 | 3534 | 1878 | 1507 | 1371 | 2089 | 1650 | 1797 | 1368 |
| Urine sediment | 1239 | 679 | 752 | 295 | 2859 | 1372 | 977 | 522 | 2092 | 1653 | 1798 | 1365 |
| Urine | 1236 | 676 | 752 | 295 | 3518 | 1999 | 1591 | 1430 | 2093 | 1653 | 1798 | 1364 |
| Vaginal swab - biochemistry | 1093 |  | 428 |  | 2275 |  | 1084 |  | 1961 |  | 1624 |  |
| Vaginal swab - microbiome | 1058 |  | 419 |  | 2134 |  | 871 |  | 1386 |  | 1200 |  |
| Placenta punches frozen |  |  | 589 |  |  |  | 1180 |  |  |  | 1688 |  |
| Placenta punches paraffin embedded |  |  | 765 |  |  |  | 1180 |  |  |  | 1672 |  |
| Cord frozen |  |  | 590 |  |  |  | 1176 |  |  |  | 1555 |  |
| Cord – paraffin embedded |  |  | 765 |  |  |  | 1175 |  |  |  | 1604 |  |
| Membranes – paraffin embedded |  |  | 764 |  |  |  | 1142 |  |  |  | 1601 |  |
| Cord whole blood |  |  | 669 |  |  |  | 1733 |  |  |  | 1614 |  |
| Cord blood spot |  |  | 668 |  |  |  | 1658 |  |  |  | 1615 |  |
| Cord blood serum |  |  | 650 |  |  |  | 1739 |  |  |  | 1615 |  |
| Cord blood plasma |  |  | 657 |  |  |  | 1730 |  |  |  | 1614 |  |
| Cord blood buffy |  |  | 659 |  |  |  | 1663 |  |  |  | 1581 |  |

TableS2: Characteristics of pregnant participants by cohort by country

|  | **Kenya** | | **Mozambique** | | **The Gambia** | | **All sites** | |
| --- | --- | --- | --- | --- | --- | --- | --- | --- |
|  | **UNS** | **TOD** | **UNS** | **TOD** | **UNS** | **TOD** | **UNS** | **TOD** |
| **Number** | **N=3450** | **N=134** | **N=2097** |  | **N=1223** | **N=28** | **N=6,770** | **N=162** |
| **Age, median (IQR)** | 26.0 (23.0, 31.0) | 26.0 (23.0, 34.0) | 23.0 (19.0, 29.0) |  | 26.0 (22.0, 31.0) | 28.0 (24.5, 32.5) | 25.0 (21.0-30.0) | 27.0 (23.0-34.0) |
| **Age at enrolment** |  |  |  |  |  |  |  |  |
| 15-19 | 245 (7.1%) | 7 (5.2%) | 554 (26.4%) |  | 153 (12.5%) | 3 (10.7%) | 952 (14.1%) | 10 ( 6.2%) |
| 20-24 | 1069 (31.0%) | 45 (33.6%) | 626 (29.9%) |  | 329 (26.9%) | 4 (14.3%) | 2,024 (29.9%) | 49 (30.2%) |
| 25-29 | 1012 (29.3%) | 26 (19.4%) | 455 (21.7%) |  | 351 (28.7%) | 11 (39.3%) | 1,818 (26.9%) | 37 (22.8%) |
| 30-34 | 710 (20.6%) | 23 (17.2%) | 270 (12.9%) |  | 217 (17.7%) | 6 (21.4%) | 1,197 (17.7%) | 29 (17.9%) |
| 35-39 | 337 (9.8%) | 21 (15.7%) | 145 (6.9%) |  | 122 (10.0%) | 2 (7.1%) | 604 ( 8.9%) | 23 (14.2%) |
| 40-44 | 71 (2.1%) | 10 (7.5%) | 42 (2.0%) |  | 43 (3.5%) | 2 (7.1%) | 156 ( 2.3%) | 12 ( 7.4%) |
| 45-49 | 5 (0.1%) | 0 (0.0%) | 5 (0.2%) |  | 4 (0.3%) | 0 (0.0%) | 14 ( 0.2%) | 0 ( 0.0%) |
| Missing | 1 (<1%) | 2 (1.5%) | 0 (0.0%) |  | 4 (0.3%) | 0 (0.0%) | 5 ( 0.1%) | 2 ( 1.2%) |
| **Marital status** |  |  |  |  |  |  |  |  |
| Never married (or single) | 212 (6.1%) | 8 (6.0%) | 906 (43.2%) |  | 22 (1.8%) | 0 (0.0%) | 1,140 (16.8%) | 8 ( 4.9%) |
| Married/ Co-habiting | 3164 (91.7%) | 118 (88.1%) | 1179 (56.2%) |  | 1196 (97.8%) | 27 (96.4%) | 5,539 (81.8%) | 145 (89.5%) |
| Separated/Divorced | 54 (1.6%) | 1 (0.7%) | 7 (0.3%) |  | 5 (0.4%) | 1 (3.6%) | 66 ( 1.0%) | 2 ( 1.2%) |
| Widowed/Missing | 20 (0.6%) | 7 (5.2%) | 5 (0.2%) |  | 0 (0.0%) | 0 (0.0%) | 25 ( 0.4%) | 7 ( 4.3%) |
| **Highest Education level** |  |  |  |  |  |  |  |  |
| None | 322 (9.3%) | 15 (11.2%) | 109 (5.2%) |  | 781 (63.9%) | 16 (57.1%) | 1,212 (17.9%) | 31 (19.1%) |
| Primary | 1812 (52.5%) | 73 (54.5%) | 704 (33.6%) |  | 193 (15.8%) | 5 (17.9%) | 2,709 (40.0%) | 78 (48.1%) |
| Secondary | 895 (25.9%) | 30 (22.4%) | 1258 (60.0%) |  | 192 (15.7%) | 6 (21.4%) | 2,345 (34.6%) | 36 (22.2%) |
| Higher | 408 (11.8%) | 9 (6.7%) | 26 (1.2%) |  | 56 (4.6%) | 1 (3.6%) | 490 ( 7.2%) | 10 ( 6.2%) |
| Missing | 13 (0.4%) | 7 (5.2%) | 0 (0.0%) |  | 1 (0.1%) | 0 (0.0%) | 14 ( 0.2%) | 7 ( 4.3%) |
| **Occupation** |  |  |  |  |  |  |  |  |
| Housewife | 1892 (54.8%) | 71 (53.0%) | 1524 (72.7%) |  | 1074 (87.8%) | 25 (89.3%) | 4,490 (66.3%) | 96 (59.3%) |
| Student | 69 (2.0%) | 5 (3.7%) | 344 (16.4%) |  | 13 (1.1%) | 0 (0.0%) | 426 ( 6.3%) | 5 ( 3.1%) |
| Professional | 248 (7.2%) | 8 (6.0%) | 61 (2.9%) |  | 20 (1.6%) | 0 (0.0%) | 329 ( 4.9%) | 8 ( 4.9%) |
| Factory | 83 (2.4%) | 3 (2.2%) | 15 (0.7%) |  | 0 (0.0%) | 0 (0.0%) | 98 ( 1.4%) | 3 ( 1.9%) |
| Large-scale agriculture | 4 (0.1%) | 0 (0.0%) | 20 (1.0%) |  | 9 (0.7%) | 0 (0.0%) | 33 ( 0.5%) | 0 ( 0.0%) |
| Market trader | 340 (9.9%) | 5 (3.7%) | 91 (4.3%) |  | 45 (3.7%) | 3 (10.7%) | 476 ( 7.0%) | 8 ( 4.9%) |
| Construction | 2 (0.1%) | 0 (0.0%) | 1 (<1%) |  | 0 (0.0%) | 0 (0.0%) | 3 ( 0.0%) | 0 ( 0.0%) |
| Business | 325 (9.4%) | 17 (12.7%) | 38 (1.8%) |  | 0 (0.0%) | 0 (0.0%) | 325 ( 4.8%) | 17 (10.5%) |
| Informal - Employment | 441 (12.8%) | 16 (11.9%) | 0 (0.0%) |  | 0 (0.0%) | 0 (0.0%) | 441 ( 6.5%) | 16 ( 9.9%) |
| Other (specify) | 31 (0.9%) | 2 (1.5%) | 0 (0.0%) |  | 61 (5.0%) | 0 (0.0%) | 130 ( 1.9%) | 2 ( 1.2%) |
| Missing | 15 (0.4%) | 7 (5.2%) | 3 (0.1%) |  | 1 (0.1%) | 0 (0.0%) | 19 ( 0.3%) | 7 ( 4.3%) |
| **Religion** |  |  |  |  |  |  |  |  |
| Muslim | 1344 (39.0%) | 42 (31.3%) | 27 (1.3%) |  | 1215 (99.3%) | 28 (100.0%) | 2,586 (38.2%) | 70 (43.2%) |
| Christian | 2082 (60.3%) | 84 (62.7%) | 2042 (97.4%) |  | 8 (0.7%) | 0 (0.0%) | 4,132 (61.0%) | 84 (51.9%) |
| Other (specify) | 11 (0.3%) | 1 (0.7%) | 26 (1.2%) |  | 0 (0.0%) | 0 (0.0%) | 37 ( 0.5%) | 1 ( 0.6%) |
| Missing | 13 (0.4%) | 7 (5.2%) | 2 (0.1%) |  | 0 (0.0%) | 0 (0.0%) | 15 ( 0.2%) | 7 ( 4.3%) |
| **% likelihood below the USAID-extreme poverty line, median (IQR)** | 2.3 (0.3, 7.4) | 2.3 (0.3, 15.4) | 5.7 (0.0, 12.9) |  | 25.2 (12.9, 28.9) | 21.4 (12.8, 25.7) | 5.7 (0.3-15.4) | 2.5 (0.4-16.8) |
| **% likelihood below the poverty line, median (IQR)** | 13.9 (4.6, 30.0) | 13.9 (4.6, 36.9) | 21.4 (7.2, 31.7) |  | 46.9 (27.9, 58.6) | 46.9 (22.2, 53.7) | 20.3 (6.1-36.9) | 17.8 (6.1-46.4) |
| **Maternal BMI, median (IQR)** | 24.0 (21.5, 27.7) | 26.6 (24.1, 30.6) | 24.2 (22.1, 27.0) |  | 22.0 (19.8, 24.7) | 21.9 (19.4, 27.9) | 23.8 (21.3-27.0) | 26.2 (23.4-30.6) |
| **Maternal BMI** |  |  |  |  |  |  |  |  |
| <18.5 | 143 (4.1%) | 0 (0.0%) | 39 (1.9%) |  | 163 (13.3%) | 3 (10.7%) | 345 ( 5.1%) | 3 ( 1.9%) |
| 18.5-24.9 | 1853 (53.7%) | 40 (29.9%) | 1172 (55.9%) |  | 778 (63.6%) | 15 (53.6%) | 3,803 (56.2%) | 55 (34.0%) |
| 25-29.9 | 908 (26.3%) | 34 (25.4%) | 661 (31.5%) |  | 205 (16.8%) | 4 (14.3%) | 1,774 (26.2%) | 38 (23.5%) |
| 30+ | 522 (15.1%) | 36 (26.9%) | 225 (10.7%) |  | 74 (6.1%) | 6 (21.4%) | 821 (12.1%) | 42 (25.9%) |
| Missing | 24 (0.7%) | 24 (17.9%) | 0 (0.0%) |  | 3 (0.2%) | 0 (0.0%) | 27 ( 0.4%) | 24 (14.8%) |
| **Parity** |  |  |  |  |  |  |  |  |
| Zero | 990 (28.7%) | 56 (41.8%) | 828 (39.5%) |  | 234 (19.1%) | 3 (10.7%) | 2,052 (30.3%) | 60 (37.0%) |
| One | 922 (26.7%) | 20 (14.9%) | 507 (24.2%) |  | 207 (16.9%) | 3 (10.7%) | 1,636 (24.2%) | 23 (14.2%) |
| Two | 639 (18.5%) | 20 (14.9%) | 336 (16.0%) |  | 179 (14.6%) | 6 (21.4%) | 1,154 (17.0%) | 25 (15.4%) |
| Three | 401 (11.6%) | 12 (9.0%) | 244 (11.6%) |  | 152 (12.4%) | 3 (10.7%) | 797 (11.8%) | 15 ( 9.3%) |
| Four | 238 (6.9%) | 9 (6.7%) | 123 (5.9%) |  | 163 (13.3%) | 5 (17.9%) | 524 ( 7.7%) | 14 ( 8.6%) |
| Five or More | 260 (7.5%) | 17 (12.7%) | 59 (2.8%) |  | 288 (23.5%) | 8 (28.6%) | 607 ( 9.0%) | 25 (15.4%) |
| **HIV status** |  |  |  |  |  |  |  |  |
| No | 3349 (97.1%) | 131 (97.8%) | 1855 (88.5%) |  | 1202 (98.3%) | 28 (100.0%) | 6,406 (94.6%) | 159 (98.1%) |
| Yes | 101 (2.9%) | 3 (2.2%) | 242 (11.5%) |  | 21 (1.7%) | 0 (0.0%) | 364 ( 5.4%) | 3 ( 1.9%) |
| Missing | 0 (0.0%) | 0 (0.0%) | 0 (0.0%) |  | 0 (0.0%) | 0 (0.0%) | 0 (0.0%) | 0 (0.0%) |
| **Village – Rural and urban index #** |  |  |  |  |  |  |  |  |
| Peri-urban | 1335 (38.7%) | 79 (59.0%) | 45 (2.1%) |  | 10 (0.8%) | 0 (0.0%) | 1,390 (20.5%) | 79 (48.8%) |
| Rural | 217 (6.3%) | 3 (2.2%) | 858 (40.9%) |  | 739 (60.4%) | 11 (39.3%) | 1,814 (26.8%) | 14 ( 8.6%) |
| Urban | 1898 (55.0%) | 51 (38.1%) | 1194 (56.9%) |  | 474 (38.8%) | 17 (60.7%) | 3,566 (52.7%) | 68 (42.0%) |
| Missing | 0 (0.0%) | 1 (0.7%) | 0 (0.0%) |  | 0 (0.0%) | 0 (0.0%) | 0 ( 0.0%) | 1 ( 0.6%) |

Table S3a: Maternal Pregnancy outcomes by cohort and by country

|  | **Kenya^#^** | | **Mozambique** | | **The Gambia^#^** | | **Total** | |
| --- | --- | --- | --- | --- | --- | --- | --- | --- |
|  | **UNS** | **TOD** | **UNS** | **TOD** | **UNS** | **TOD** | **UNS** | **TOD** |
| **Number of women with pregnancy outcome data** | 2592 | 114 | 1861 |  | 1154 | 24 | 5607 | 138 |
| Miscarriages (<20 weeks) | 5 (0.2%) |  | 17 (0.9%) |  | 12 (1.0%) |  | 34 (0.6%) |  |
| Maternal hypertension | 490 (18.9%) | 84 (73.7%) | 288 (15.5%) |  | 335 (29.0%) | 14 (58.3%) | 1113 (19.9%) | 98 (71.0%) |
| Maternal gestational hypertension | 377 (14.5%) | 52 (45.6%) | 267 (14.4%) |  | 234 (20.2%) | 5 (20.8%) | 878 (15.7%) | 57 (41.3%) |
| Maternal chronic hypertension | 112 (4.3%) | 30 (26.3%) | 45 (2.4%) |  | 131 (11.4%) | 10 (41.7%) | 288 (5.1%) | 40 (29.0%) |
| Maternal preeclampsia | 132 (5.1%) | 62 (54.4%) | 51 (2.7%) |  | 147 (12.7%) | 8 (33.3%) | 330 (5.9%) | 70 (50.7%) |
| Missing maternal hypertension information | 893 (34.5%) | 20(17.5%) | 81 (4.4%) |  | 309 (26.8%) | 6 (25.0%) | 1283 (22.8%) | 26 (18.8%) |
| Maternal admissions to ICU | 0 |  | 4 |  | 1 |  | 5 |  |
| Maternal deaths | 6 |  | 0 |  | 3 |  | 9 |  |
| Missing pregnancy outcome | 5 (0.1%) | 2 | 1 (0.1%) |  | 2 (0.1%) | 1 | 8 (0.1%) | 3 |

Table S3b: Infant outcomes by cohort and by country

|  | **Kenya** | | **Mozambique** | | **The Gambia** | | **Total** | |
| --- | --- | --- | --- | --- | --- | --- | --- | --- |
|  | **UNS** | **TOD** | **UNS** | **TOD** | **UNS** | **TOD** | **UNS** | **TOD** |
| **Number of children with birth outcome data (total birth)** | 2633 | 121 | **1863** |  | **1172** | **25** | **5668** | **146** |
| Number of singletons | 2541 (96.5%) | 107 (88.4%) | 1827 (98.1%) |  | 1112 (94.9%) | 23 (92.0%) | 5480 (96.7%) | 130 (89.0%) |
| Number of twins | 92 (3.5%) | 14 (11.6%) | 36 (1.9%) |  | 60 (5.1%) | 2 (8.0%) | 188 (3.3%) | 16 (11.0%) |
| **Vital status at birth** |  |  |  |  |  |  |  |  |
| Number of livebirths | 2579 (97.9%) | 103 (85.1%) | 1800 (96.7%) |  | 1105 (94.3%) | 21 (84.0%) | 5484 (96.8%) | 124 (84.9%) |
| Number of stillbirths (>/=20 weeks) | 46 (1.7%) | 16 (13.2%) | 61 (3.3%) |  | 58 (5.9%) | 3 (12.0%) | 165 (2.9%) | 19 (13.0%) |
| Missing birth outcome | 8 (0.1%) | 2 (1.7%) | 2 (0.1%) |  | 9 (0.8%) | 0 (0.0%) | 19 (0.3%) | 2 (1.4%) |
| **Mode of delivery** |  |  |  |  |  |  |  |  |
| Unassisted vaginal/Cephalic | 2073 (78.7%) | 83 (68.6%) | 1387 (74.4%) |  | 937 (79.9%) | 19 (76.0%) | 4397 (77.6%) | 102 (69.9%) |
| Operative vaginal | 1 (<1%) | 0 (0.0%) | 138 (7.4%) |  | 13 (1.1%) | 0 (0.0%) | 152 (2.7%) | 0 (0.0%) |
| Vaginal breech | 12 (0.5%) | 0 (0.0%) | 129 (6.9%) |  | 5 (0.4%) | 0 (0.0%) | 146 (2.6%) | 0 (0.0%) |
| Caesarean section | 385 (14.6%) | 34 (28.1%) | 194 (10.4%) |  | 34 (2.9%) | 0 (0.0%) | 613 (10.8%) | 34 (23.3%) |
| Missing mode of delivery | 162 (6.2%) | 4 (3.3%) | 15 (0.8%) |  | 183 (15.6%) | 6 (24.0%) | 360 (6.4%) | 10 (6.8%) |
| **Birth weight (Total births)** |  |  |  |  |  |  |  |  |
| Median (IQR) | 3010.0 (2705.0, 3292.5) | 2843.8 (2347.5, 3160.0) | 3092.5 (2800, 3390) |  | 3000.0 (2717.2, 3311.2) | 3000.0 (2302.5, 3200.0) | 3025.0 (2740.0, 3310.0) | 2863.8 (2347.5, 3165.0) |
| ~~Missing birthweight~~ |  |  |  |  |  |  |  |  |
| **Birth weight categories** |  |  |  |  |  |  |  |  |
| <2500g | 312 (11.8%) | 36 (29.8%) | 198 (10.6%) |  | 116 (9.9%) | 5 (20.0%) | 626 (11.0%) | 41 (28.1%) |
| 2500g to 4000g | 1939 (73.6%) | 73 (60.3%) | 1615 (86.7%) |  | 835 (71.2%) | 13 (52.0%) | 4389 (77.4%) | 86 (58.9%) |
| >4000g | 45 (1.7%) | 1 (0.8%) | 31 (1.7%) |  | 9 (0.8%) | 0 (0.0%) | 85 (1.5%) | 1 (0.7%) |
| Missing birth weight | 337 (12.8%) | 11 (9.1%) | 19 (1.0%) |  | 212 (18.1%) | 7 (28.0%) | 568 (10.0%) | 18 (12.3%) |
| **Gestational age at delivery** | 2579 (97.9%) | 115 (95.0%) | 1855 (99.6%) |  | 1129 (96.3%) | 23 (92.0%) | 5563 (98.1%) | 138 (94.5%) |
| Median (IQR) | 39.1 (37.3, 40.6) | 38.0 (36.0, 39.1) | 39.6 (38.0, 40.9) |  | 39.0 (37.1, 40.4) | 38.7 (34.4, 40.1) | 39.1 (37.6, 40.6) | 38.0 (35.9, 39.1) |
| Missing GA | 54 (2.1%) | 6 (5.0%) | 8 (0.4%) |  | 43 (3.7%) | 2 (8.0%) | 105 (1.9%) | 8 (5.5%) |
| **Preterm birth categories (total births)** |  |  |  |  |  |  |  |  |
| Extremely preterm: <28+0 weeks | 31 (1.2%) | 5 (4.1%) | 23 (1.2%) |  | 21 (1.8%) | 1 (4.0%) | 75 (1.3%) | 6 (4.1%) |
| Very preterm: 28+0 - 31+6 | 55 (2.1%) | 5 (4.1%) | 31 (1.7%) |  | 33 (2.8%) | 2 (8.0%) | 119 (2.1%) | 7 (4.8%) |
| Moderate preterm: 32+0 - 33+6 | 91 (3.5%) | 6 (5.0%) | 36 (1.9%) |  | 29 (2.5%) | 2 (8.0%) | 156 (2.8%) | 8 (5.5%) |
| Late preterm: 34+0 - 36+6 | 333 (12.6%) | 22 (18.2%) | 171 (9.2%) |  | 144 (12.3%) | 3 (12.0%) | 648 (11.4%) | 25 (17.1%) |
| Total preterm (<37+0 weeks) | 510 (19.4%) | 38 (31.4%) | 261 (14.0%) |  | 227 (19.4%) | 8 (32.0%) | 998 (17.6%) | 46 (31.5%) |
| **Size for gestational age** |  |  |  |  |  |  |  |  |
| Severely Small for Gestational Age (<3rd centile) | 166 (6.3%) | 11 (9.1%) | 142 (7.6%) |  | 78 (6.7%) | 3 (12.0%) | 386 (6.8%) | 14 (9.6%) |
| Small for Gestational Age (3^rd^ - 10^th^ centile) | 260 (9.9%) | 13 (10.7%) | 203 (10.9%) |  | 114 (9.7%) | 1 (4.0%) | 577 (10.2%) | 14 (9.6%) |
| Appropriate for Gestational Age (10^th^ to 90^th^ centile) | 1544 (58.6%) | 66 (54.5%) | 1313 (70.5%) |  | 608 (51.9%) | 13 (52.0%) | 3465 (61.1%) | 79 (54.1%) |
| Large for Gestational Age (>90th centile) | 167 (6.3%) | 10 (8.3%) | 96 (5.2%) |  | 68 (5.8%) | 1 (4.0%) | 331 (5.8%) | 11 (7.5%) |
| Extremely Large for Gestational Age (>97th centile) | 77 (2.9%) | 3 (2.5%) | 55 (3.0%) |  | 38 (3.2%) | 0 (0.0%) | 170 (3.0%) | 3 (2.1%) |
| Missing size for gestational age | 419 (15.9%) | 18 (14.9%) | 54 (2.9%) |  | 266 (22.7%) | 7 (28.0%) | 739 (13.0%) | 25 (17.1%) |
| **Neonatal admission to neonatal unit** |  |  |  |  |  |  |  |  |
| Yes | 52 (2.0%) | 5 (4.2%) | 58 (3.1%) |  | 15 (1.3%) | 0 (0.0%) | 125 (2.2%) | 5 (3.4%) |
| Missing admission to neonatal unit | 440 (16.7%) | 23 (19.0%) | 80 (4.3%) |  | 290 (24.7%) | 9 (36.0%) | 812 (14.3%) | 32 (21.9%) |
