# Supplementary material for: PREgnancy Care Integrating translational Science, Everywhere (PRECISE): a prospective cohort study of African pregnant and non-pregnant women to investigate placental disorders – cohort profile
Source: BMJ Open. 2025 May 11;15(5):e091831. doi: 10.1136/bmjopen-2024-091831 (PMC12067852; doi:10.1136/bmjopen-2024-091831)
Supplement: Supplementary file 6 [file bmjopen-2024-091831.reviewer_comments.pdf]

# PEER REVIEW HISTORY

BMJ Open publishes all reviews undertaken for accepted manuscripts. Reviewers are asked to complete a checklist review form and are provided with free text boxes to elaborate on their assessment. These free text comments are reproduced below.

## ARTICLE DETAILS

### Title (Provisional)

Cohort Profile: PREgnancy Care Integrating translational Science, Everywhere (PRECISE), a prospective cohort study of African pregnant and non-pregnant women to investigate placental disorders

### Authors

Craik, Rachel; Akuze, Joseph; Volvert, Marie-Laure Christane; Blencowe, Hannah; Mukhanya, Moses; Makanga, Prestige Tatenda; Tchavana, Corssino; Moore, Sophie; Vala, Anifa; Koech, Angela; Tribe, Rachel M.; Noble , Alison; Bah, Baboucar; D'Alessandro, Umberto; Vidler, Marianne; Tu, Domena; Maculuve, Sonia; Wanje , Onesmus; Idris, Yahaya; Mwashigadi, Grace; Ochieng , Marvin; Filippi, Veronique; Roca, Anna; Magee, Laura A.; Poston, Lucilla; Mistry, Hiten; Bah, Yorro; Li, Jing; Temmerman, Marleen; Sevene, Esperanca; Jah, Hawanatu; Mwadime , Emily; Barratt, Ben; Papageorghiou, Aris T; Makacha, Liberty; Quimice, Lazaro; Touray, Fatima; Salisbury, Tatiana; Kongira, Fatoumata; von Dadelszen, Peter; and the PRECISE Network , ,

---

## VERSION 1 - REVIEW

---

| | |
|---|---|
| **Reviewer** | **1** |
| **Name** | **Karmakar, Subhradip** |
| **Affiliation** | **All India Institute of Medical Sciences, India, Biochemistry and Mocular Medicine** |
| **Date** | **20-Dec-2024** |
| **COI** | **None** |

---

Cohort Profile: PREgnancy Care Integrating translational Science, Everywhere (PRECISE), a

prospective cohort study of African pregnant and non-pregnant women to investigate placental

disorders.

The authors established “The PRECISE Network in Sub-Saharan province to investigate specific placental disorders (pregnancy hypertension, preterm birth, fetal growth restriction, and stillbirth. The authors recruited around 6932 females and 1825 non-pregnant women.

This is an excellent study and surely is well-needed for the Sub-African province, given the high prevalence of placental disorders there that are largely undocumented so far.

Some concerns need to be addressed :

Abstract:

1. The abstract lacks information on how the samples in such large numbers will be labeled, bookmarked, and stored or may be retrieved at a later date when required

2. The information on non-pregnant women needs clarification of whether these are married non-pregnant or unmarried non-pregnant, as these two groups will have different health parameters and physiology. Do non-pregnant married women have other health issues for which they failed to conceive? I see some information in one of the tables but not much in the text.

Main Manuscript :

The manuscript is written well and presently elegantly with a nice, detailed description. Undoubtedly, very important study with a lot of potential. Some concerns are listed :

1. I didn't find any description of the subjects screened for TORCH infection, which is common during pregnancy.

2. Give an estimate of how many researchers have so far used the samples or data and what meaningful results were delivered

3. The authors mentioned that they first used BAOBAB for data management, following which they moved to an Open Source system. Throughout this process and migration, has the data been managed in encrypted form to maintain privacy? Who handles and manages the patient data besides Kings College and u of BC? Is there any local management team on the ground? Kindly elaborate

4. As per Annex C ,it seems that the sample collection at the Gambia center is lagging behind the other two(Kenya and Mozambique) ? Is there any specific reason for these comparatively low numbers regarding enrolled subjects or other technical issues?

5. It is essential that the authors mention how they are storing the biological samples. Freezers? Lig N2 ? since specimens meant for different assays need to be stored at specific locations and temperatures. It would be helpful to have a table /Annex for this. Sooner, the sample numbers will increase further, and cataloging at this stage will be beneficial.

6. It seems the ToD cohort included subjects not enrolled in the UNS cohort. Are these samples from ToD also collected and stored with the ANS samples? How many first-time emergency cases with HDP or pre-eclampsia were not under any study groups?

7. I could have missed out, but it seems economic status or household income ( measures as income /month or annual income) was not listed for the subjects. Given that socioeconomic

status significantly impacts maternal and child health, this information will be helpful. Kindly comment

8. I see that the authors mentioned successfully setting up the biorepository and PRECISE database, which is an extraordinary achievement. I am just curious if the local manpower is adequately trained to handle the precious samples and do meaningful biology on this. There is no description of capacity building and training programs, and unless this is addressed and achieved, the full potential of this cohort will remain underutilized.

9. Is there any financial compensation for the subjects enrolled in this study or for self-volunteers?

10. Descriptions of Medications, physicians' costs, and other interventions are not well discussed. It will be good to add a note on this.

Overall, it is a powerful study with a lot of potential. Very high-level management is needed to use the PRECISE study at this potential to benefit the local population. The sample and data sharing part need more transparency and elaborate description. The rules and compliances need to be discussed so that researchers outside this study group can access these study and sample materials with due permission.

---

| | |
|---|---|
| **Reviewer** | **2** |
| **Name** | **Pinto, Pedro Viana** |
| **Affiliation** | **Centro Hospitalar São João, Porto, Portugal** |
| **Date** | **29-Dec-2024** |
| **COI** | **None** |

---

The PRECISE study is a prospective cohort of African (sub-Saharan) women. It is a well described and conducted study, aiming to report highly relevant outcomes.

The study sumbitted is a cohort profile, aiming to report what has already been done ando found in the cohort.

Some points could be more adequately reported.

Regarding the time of disease cohort, what was the reason for excluding patients from Mozambique? And why the differences in recruitment in the WRA group between the countries?

Something interesting to be reported is that the majority of women in Kenya and Mozambique live in urban areas (and peri-urban); only in Gambia the majority lives in rural areas; is this reprresentative of the populations?

The low rate of miscarriages reported is due to the recruitment? (majority of women recruited with median gestational age >19 weeks). Table 5a, maternal hypertension includes chronic and gestational? Preeclampsia also included?

18% of premature deliveries is a high number - should the authors go trhough it in more detail or is it not relevant in this cohort profile?

Page 14, line 19, sentence shouldn't start with a number.

Minor mistakes in english should be addressed (eg. page 10, line 33 with is wrtten twice; line 45 after Kenya "and" seemes mistakenly written)

---

## VERSION 1 - AUTHOR RESPONSE

Reviewer: 1

Dr. Subhradip Karmakar, All India Institute of Medical Sciences, India

Comments to the Author:

Cohort Profile: PREgnancy Care Integrating translational Science, Everywhere (PRECISE), a prospective cohort study of African pregnant and non-pregnant women to investigate placental disorders.

The authors established "The PRECISE Network in Sub-Saharan province to investigate specific placental disorders (pregnancy hypertension, preterm birth, fetal growth restriction, and stillbirth. The authors recruited around 6932 females and 1825 non-pregnant women. This is an excellent study and surely is well-needed for the Sub-African province, given the high prevalence of placental disorders there that are largely undocumented so far.

Some concerns need to be addressed :

Abstract:

1. The abstract lacks information on how the samples in such large numbers will be labeled, bookmarked, and stored or may be retrieved at a later date when required

These details has been added, in brief.

2. The information on non-pregnant women needs clarification of whether these are married non-pregnant or unmarried non-pregnant, as these two groups will have different health parameters and physiology. Do non-pregnant married women have other health issues for which they failed to conceive? I see some information in one of the tables but not much in the text.

Thanks for the suggestion. We have added text to the Participants section of the Abstract. In addition, we have added some more detail to the Methods section.

Main Manuscript :

The manuscript is written well and presently elegantly with a nice, detailed description. Undoubtedly, very important study with a lot of potential. Some concerns are listed :

Thank you for these kind comments.

1. I didn't find any description of the subjects screened for TORCH infection, which is common during pregnancy.

Screening for TORCH infections is not routine in these sites. We plan to use our biorepository to undertake these (and other infectious disease) assays in future research.

2. Give an estimate of how many researchers have so far used the samples or data and what meaningful results were delivered

We have added a Biological Samples sub-heading to the results section. We would ask that we not be required to provide detailed results of these studies as most have yet to be published and it would reduce our ability to do so.

3. The authors mentioned that they first used BAOBAB for data management, following which they moved to an Open Source system. Throughout this process and migration, has the data been managed in encrypted form to maintain privacy? Who handles and manages the patient data besides Kings College and u of BC? Is there any local management team on the ground? Kindly elaborate

We have clarified in the text under the Data and Sample Management sub-heading. All data and samples are the property of the country teams in which the participants resided during their participation. No identifiers are ever shared outside the country – encrypted data are shared. Each study maintains an updated version of their dataset, and any queries are run through them; updates are shared between the country database and the linked database with data from the three countries (currently at KCL). Women are identified by a unique study ID that is shared between the clinical database and laboratory information system. BAOBAB and OpenSpecimen have been used solely for specimen tracking. Social determinant and clinical data have been maintained through ODK-X (The Gambia and Kenya) and REDCap (Mozambique).

4. As per Annex C ,it seems that the sample collection at the Gambia center is lagging behind the other two(Kenya and Mozambique) ? Is there any specific reason for these comparatively low numbers regarding enrolled subjects or other technical issues?

There are no clear reasons for the lower number in sample in The Gambia; however, the disruption caused by the pandemic did appear to disrupt that team more than the other two countries.

5. It is essential that the authors mention how they are storing the biological samples. Freezers? Lig N2 ? since specimens meant for different assays need to be stored at specific locations and temperatures. It would be helpful to have a table /Annex for this. Sooner, the sample numbers will increase further, and cataloging at this stage will be beneficial.

We have added more details to the Biological samples sub-heading. We have added the reference to the published PRECISE biobanking protocol that provides the details.

6. It seems the ToD cohort included subjects not enrolled in the UNS cohort. Are these samples from ToD also collected and stored with the ANS samples? How many first-time emergency cases with HDP or pre-eclampsia were not under any study groups?

That is true. In The Gambia and Kenya, the ToD cohort were recruited if they were referred into a study site with evidence of either pregnancy hypertension or fetal death.

The details are provided in the Flow Chart (Fig 1). Their samples were also collected and stored with the UNS samples.

7. I could have missed out, but it seems economic status or household income ( measures as income /month or annual income) was not listed for the subjects. Given that socioeconomic status significantly impacts maternal and child health, this information will be helpful. Kindly comment

Rather than use household income, we have chosen to use the Poverty Probability Index that standardises against local costs of living (https://www.povertyindex.org/about-ppi). This is our marker of socioeconomic status. We have two metrics (presented in Table 4), the probability of extreme poverty (USAID poverty line) and the probability of poverty. Detailed health economics analyses are underway.

8. I see that the authors mentioned successfully setting up the biorepository and PRECISE database, which is an extraordinary achievement. I am just curious if the local manpower is adequately trained to handle the precious samples and do meaningful biology on this. There is no description of capacity building and training programs, and unless this is addressed and achieved, the full potential of this cohort will remain underutilized.

Our Network partners were chosen with care. The three clinical sites have international reputations for stellar laboratory-based research in their settings, primarily built around infectious disease-related work. In addition, Ms Craik, the first author of this paper, and Ms Donna Russell, who both have considerable experience setting up biorepositories in LMICS and standardising procedures, travelled repeatedly to the sites to help them establish their pathways dealing with (for the sites) novel biological samples such as placentae. Our QC results reflect the excellent work of our teams. More details about training can also be found here https://doi.org/10.1186/s12978-020-0874-7

9. Is there any financial compensation for the subjects enrolled in this study or for self-volunteers?

Women were refunded travel costs and provided with food and drink when they attended a project-specific encounter.

10. Descriptions of Medications, physicians' costs, and other interventions are not well discussed. It will be good to add a note on this.

These questions and analyses were part of the PRECISE-DYAD project, which has been described separately (Craik R, *et al*. The PRECISE-DYAD protocol: linking maternal and infant health trajectories in sub-Saharan Africa. Wellcome Open Res. 2024 Apr 5;7:281. doi: 10.12688/wellcomeopenres.18465.2. PMID: 38779418; PMCID: PMC11109552). The results will be published in due course.

Overall, it is a powerful study with a lot of potential. Very high-level management is needed to use the PRECISE study at this potential to benefit the local population. The sample and data sharing part need more transparency and elaborate description. The rules and compliances need to be discussed so that researchers outside this study group can access these study and sample materials with due permission.

This is described in some detail under the Collaboration sub-heading. We added a link to the Network website where the process is provided in full detail.

Reviewer: 2

Dr. Pedro Viana  Pinto, Centro Hospitalar São João, Porto, Portugal

Comments to the Author:

The PRECISE study is a prospective cohort of African (sub-Saharan) women. It is a well described and conducted study, aiming to report highly relevant outcomes.

Thank you.

The study sumbitted is a cohort profile, aiming to report what has already been done ando found in the cohort.

Some points could be more adequately reported.

Regarding the time of disease cohort, what was the reason for excluding patients from Mozambique? And why the differences in recruitment in the WRA group between the countries?

Each country developed its own version of the protocol in discussion with their teams (for logistics) and local ethics boards. In this instance, it was decided that Mozambique would not recruit a separate time of disease cohort.

Something interesting to be reported is that the majority of women in Kenya and Mozambique live in urban areas (and peri-urban); only in Gambia the majority lives in rural areas; is this reprresentative of the populations?

These results are representative of the areas in which we were working. Our health geography team would place the peri-urban families in the 'rural' group were we to dichotomise the assignment.

The low rate of miscarriages reported is due to the recruitment? (majority of women recruited with median gestational age >19 weeks). Table 5a, maternal hypertension includes chronic and gestational? Preeclampsia also included?

Yes, most miscarriages would have occurred before women booked for antenatal care. In addition, we have chosen the 1950 WHO 20+0 week/500g threshold for a 'birth', meaning that any loss ≥20+0 was counted as either a stillbirth or neonatal death.

At the time of submitting the original manuscript, we had some residual uncertainty about how to assign the diagnoses for women whose pregnancies were complicated by the various hypertensive disorders of pregnancy. These data have now been provided and updated with confidence.

18% of premature deliveries is a high number - should the authors go trhough it in more detail or is it not relevant in this cohort profile?

We would ask that we can present these data and analyses separately, as this is the focus of Professor Tribe's sub-grant from the MRC. We are confident in our dating and, as with pre-eclampsia, are surprised by the burden of pregnancy complications borne by women in these settings. We hope that the PRECISE data and biological repositories will elucidate the 'whys' of this situation and provide evidence to support novel context-relevant interventions.

Page 14, line 19, sentence shouldn't start with a number.

Corrected, thank you.

Minor mistakes in english should be addressed (eg. page 10, line 33 with is wrtten twice; line 45 after Kenya "and" seemes mistakenly written)

Thank you.

---

## VERSION 2 - REVIEW

---

**Reviewer** **1**

**Name** **Karmakar, Subhradip**

**Affiliation** **All India Institute of Medical Sciences, India, Biochemistry and Mocular Medicine**

**Date** **28-Mar-2025**

**COI**

---

The authors have successfully addressed all the queries and improved the manuscript substantially

---

**Reviewer** **2**

**Name** **Pinto, Pedro Viana**

**Affiliation** **Centro Hospitalar São João, Porto, Portugal**

**Date** **07-Apr-2025**

**COI**

---

I am happy with the reviews made
